# Quantum Engineering of Atomically Smooth Single-Crystalline Silver Films

**DOI:** 10.1038/s41598-019-48508-3

**Published:** 2019-08-22

**Authors:** Ilya A. Rodionov, Aleksandr S. Baburin, Aidar R. Gabidullin, Sergey S. Maklakov, Sven Peters, Ilya A. Ryzhikov, Alexander V. Andriyash

**Affiliations:** 1 FMN Laboratory, Bauman Moscow State Technical University, Moscow, Russian Federation; 2 Dukhov Research Institute of Automatics, Moscow, Russian Federation; 3 SENTECH Instruments GmbH, Berlin, Germany; 4 Institute for Theoretical and Applied Electromagnetics RAS, Moscow, Russian Federation

**Keywords:** Materials for optics, Nanophotonics and plasmonics, Synthesis and processing, Sensors and biosensors, Superconducting properties and materials

## Abstract

There is a demand for ultra low-loss metal films with high-quality single crystals and perfect surface for nanophotonics and quantum information processing. Many researches are devoted to alternative materials, but silver is by far theoretically the most preferred low-loss material at optical and near-IR frequencies. Usually, epitaxial growth is used to deposit single-crystalline silver films, but they still suffer from unpredictable losses and well-known dewetting effect that strongly limits films quality. Here we report the two-step approach for e-beam evaporation of atomically smooth single-crystalline metal films. The proposed method is based on the thermodynamic control of film growth kinetics at atomic level, which allows depositing state-of-art metal films and overcoming the film-surface dewetting. Here we use it to deposit 35–100 nm thick single-crystalline silver films with the sub-100pm surface roughness and theoretically limited optical losses, considering an ideal material for ultrahigh-Q nanophotonic devices. Utilizing these films we experimentally estimate the contribution of grain boundaries, material purity, surface roughness and crystallinity to optical properties of metal films. We demonstrate our «SCULL» two-step approach for single-crystalline growth of silver, gold and aluminum films which open fundamentally new possibilities in nanophotonics, biotechnology and superconductive quantum technologies. We believe it could be readily adopted for the synthesis of other extremely low-loss single-crystalline metal films.

## Introduction

Unique large-scale optoelectronic devices utilizing plasmonic effects for near-field manipulation, amplification and sub-wavelength integration open new frontiers in nanophotonics, quantum optics and quantum information science^1–15^. However, ohmic losses in metals are still a big challenge on the way towards a variety of useful plasmonic devices^14–21^. Many researchers have devoted extensive efforts in clarifying the comprehensive influence of metal film properties on overall losses to develop a high performance material platform^14–19^. Single-crystalline platform has the potential to alleviate this problem by eliminating material-induced scattering losses^3,4^ and nanoscale structure definition impact^15–18^. Silver (Ag) is by far potentially the best plasmonic metal at optical and near-IR frequencies^4,15–19^, when it comes to the optical loss, silver is still superior to all new plasmonic materials, including graphene^17^. On the other hand, silver is one of the most challenging metals for single-crystalline film growth^18–22^. Moreover, because of silver nature, an epitaxial growth of sub-50 nm thick and ultrathin films is impeded without using loosy wetting underlayers^23–26^. Most of the reported single-crystalline silver film growth methods rely on molecular beam epitaxy (MBE)^19,27^ or physical vapor deposition (PVD)^18^. It was demonstrated that Ag epitaxial films can be engineered to have almost atomic smoothness and significantly lower optical losses in the 1.8–2.5 eV range^19^ than widely cited Johnson and Christy (JC) data^28^. Here we report on a two-step PVD growth approach to obtain atomically smooth single-crystalline metal films, which is the result of a detailed study on the growth mechanism^29–31^. Our approach provides the single-crystalline metal films growth on non-ideally lattice-matched substrates without underlayers using a high vacuum electron-beam evaporator. It guarantees simultaneously high crystallinity and purity, atomically smooth surface over a centimetre-scale area, reproducible run-to-run film thickness (down to 35 nm), the unique optical properties and SPP propagation length, and thermodynamic stability. The process is facile, inexpensive and fast (high deposition rate) compared to MBE technique, compatible with lithography and etch nanoscale features definition, and reproducible in a standard cleanroom environment. In addition, it can be effectively applied to various metals such as silver, gold, and aluminum, which are widely used metals in quantum optics and quantum information science.

**Figure 1:**
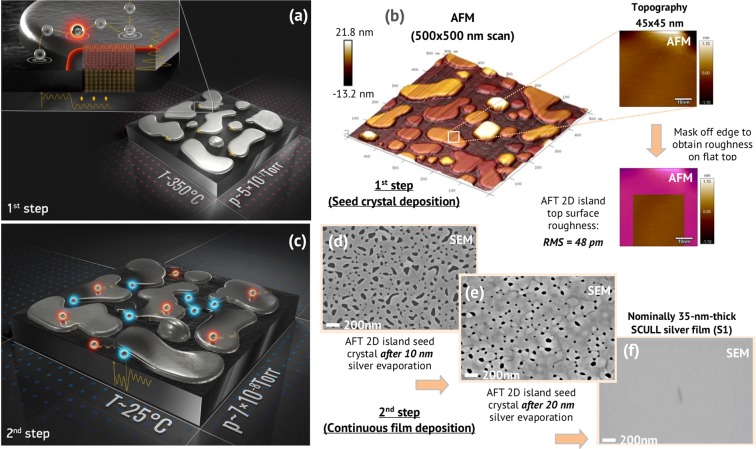
Two-step deposition of single-crystalline silver films. (**a**) In the first step, an AFT 2D. Ag (111) seed crystal is deposited under 350 °С temperature. (**b**) Atomic force microscopy (AFM) scan of AFT 2D Ag (111) islands deposited on a Si (111) substrate. Most of the AFT 2D Ag(111) islands have atomically flat top surface with an RMS roughness less than 50 pm. In the second step, the process is stopped and the substrate is cooled down to 25 °С followed by additional silver evaporation until continuous silver film is formed. (**c**) SEM images illustrate film morphology evolution during the second step after nominally 10 nm (**d**) and 20 nm (**e**) silver evaporation on a AFT 2D seed crystal at 25 °С. (**f**) SEM image of nominally 35-nm-thick single-crystalline film. The defect on the film surface is purposefully created (by electron beam burning) to facilitate focusing on the atomically smooth surface.

Figure 1 illustrates the basic principles of atomically smooth single-crystalline silver films two-step deposition process. In the first step, a seed crystal consisting of strained two-dimensional islands with atomically flat top surface (AFT 2D islands) is grown on a substrate (see Supplementary Information for details) under 350 °С. In the second step, the deposition is stopped and the substrate is cooled down to 25 °С in the same vacuum cycle to prevent a well-known dewetting effect and three-dimensional growth leading to subsequent film imperfections. Then, more silver is deposited on AFT 2D seed at 25 °С until a continuous film is formed. Following film annealing under elevated temperature (higher than first step) can reduce growth defects density improving crystalline structure and surface roughness. We believe that simultaneous improvement in film characteristics relies on the combination of two mixed evaporation modes combined with the AFT 2D islands growth self-controlled by quantum size effects. With this dual-phase experimental nature in mind as well as improved film parameters, we name our deposition process the «SCULL» (Single-crystalline Continuous Ultra-smooth Low-loss Low-cost).

### The SCULL process

Consider a silver (111) film on silicon (111), which is a well-known substrate for epitaxial Ag growth. To quantitatively estimate an influence of substrate crystalline structure on films quality, we additionally deposit the SCULL silver films on widely used silicon (100), silicon (110), and mica substrates. Nominally 35-nm-thick single-crystalline silver films were evaporated using the SCULL process (base pressure 3 × 10^−8^ Torr, see Supplementary Information) on different substrates (Table 1), which are the thinnest PVD single-crystalline silver films than those reported previously^18,32,33^. On the other hand, the SCULL process has no fundamental limitations in a thicker film synthesis, which is crucial for applications sensitive to SPP substrate absorption. To demonstrate this, we deposit nominally 70-nm-thick (S4) and 100-nm-thick Ag (111) films (S5) on Si (111) substrates. All the films are continuous, without voids and pits, and have an atomically smooth surface over 15 × 15 mm^2^ sample area.

**Table 1:** Thickness, surface roughness and microstructure of SCULL Ag films as a function of substrate type.

Substrate	Measured thickness, [nm]	Crystalline structure	Average grain size, [nm]	Rocking curve Ag peak, FWHM [°]	AFM RMS roughness, [nm]	Sample
Mica	35	Single-crystalline	no grains	not measured	0.35	M1
Si (111)	37	Single-crystalline	no grains	0.325	0.09	S1
Si (100)	39	Single-crystalline	no grains	0.829	0.28	S2
Si (110)	39	Single-crystalline	no grains	0.831	0.37	S3
Si (111)	68	Single-crystalline	no grains	0.221	0.36	S4
Si (111)	99	Single-crystalline	no grains	0.368	0.43	S5
Quartz	107	Nanocrystalline	less than 20	not measured	2.18	NC
Quartz	100	Polycrystalline	50	not measured	2.34	PC
Quartz	98	Polycrystalline	more than 500	not measured	2.22	PCBG

AFM RMS roughness was determined from scans over a 2.5 × 2.5 μm^2^ area. SP RMS roughness was determined from scans over a 20-μm length.

According to the epitaxy theory and long-term experience a microstructure, growth mode and morphology are mostly governed by kinetic effects at high deposition rates (non-equilibrium experimental conditions). In this case single-crystalline films can be deposited in a smooth Frank-van der Merwe (layer-by-layer) growth mode^34^, when the surface free energy of the substrate (E_sub_) is higher than or equal to the sum of the surface free energies, *E*_*sub*_ ≥ *E*_*f*_ + *E*_*int*_, for the film (E_f_) and the interface (E_int_). Then, it is energetically favorable the film to cover the substrate completely to eliminate the contribution of the high substrate surface energy. The fundamental idea of our process involves quantum engineering of the AFT 2D seed crystal of a given metal (1st step), which is «frozen» at the optimum point of the growth process, followed by dramatic shift of film growth kinetics (2nd step) allowing the lateral spreading of the crystal seed until the perfect continuous film is formed. Three key features (Fig. 1a,b) have to be provided at the first process step: the 2D growth of islands with atomically flat top surface, the macroscopic control of thickness and microstructure of AFT 2D islands and the well-defined strain accumulated in islands at the optimum point. 2D growth can be guaranteed by interlayer mass transport control, which is the delicate balance between the adatoms surface diffusion (*D ~ temperature*) and the flux (*F ~ deposition rate*), the growing islands state (density, size, shape and strain), the surface diffusion (*E*_*D*_) and the step-edge Ehrlich-Schwoebel (*E*_*S*_) barriers for adatoms to descend (downward transport) or ascend (upward transport) the edges of the growing islands.

In order to ascend (descend) an island, the adatoms arriving on a substrate (island) surface may try several times (with the hopping rate *ν* = *ν*_0_ *exp*(−*E*_*S*_*/k*_*B*_*T*)) to move over the edge barrier *∆E* = *E*_*S*_* − E*_*D*_. Based on previously reported data^35,36^, we experimentally determine the ranges of adatoms surface diffusion (280–420 °С temperature), adatoms flux (0.5–10 Å s^−1^ deposition rates) and islands state (1–25 nm thickness) for the layer-by-layer AFT 2D Ag (111) islands growth at the first process step. Above the certain islands state (size, interface area, strain) the sum of the islands surface free energies *E*_*f*_ + *E*_*int*_ becomes higher than the substrate surface free energy *E*_*sub*_, leading to three-dimensional growth. Moreover, increasing the islands dimensions without reducing the adatom surface diffusion *D* results in an enhanced hopping rate *ν* of adatoms visiting the step edges and, thus, an increased upward mass transport. Thus, there is the optimum point of the process, when the AFT 2D Ag (111) islands seed deposition has to be stopped.

An electronic growth model^36,37^ based on the quantum size effects can explain the second key feature of AFT 2D islands (self-controlled thickness and crystalline structure over a large area). According to the electronic growth model, growing AFT 2D Ag (111) islands are considered as an electron gas, which is confined to a 2D quantum well that is as wide as the thickness of the silver islands^38,39^. The energy oscillates as a function of the island thickness (quantum well width), resulting in island thickness quantization (Fig. 1a, inset). Top silver layers of the islands grow under a homoepitaxial regime in the presence of these small energy oscillations, resulting in a quantized island thickness with the silver monolayer accuracy^36,37,40^. Together with the layer-by-layer 2D growth mode it enables formation of the two-dimensional Ag (111) islands with atomically flat top surface and provides precise control of the islands thickness over macro scale area, even in the presence of typical PVD process deviations.

The third key feature of AFT 2D Ag (111) seed crystal is an energy accumulation in islands which is induced by strained growth under elevated temperature on the substrate with different lattice constants. The accumulated strain affects the growth kinetics at the second process step by lowering the *E*_*S*_ barrier for the adatom surface diffusion. Strained growth is induced by the onset of defects: screw dislocation influence and spiral growth which becomes stronger with thickness increase. That is why the AFT 2D seed thickness has to be optimized to provide a dislocation-free crystalline lattice growth of Ag (111) islands, on the one hand, and the ultimate initial strain accumulation, on the other. As the result of the first step the AFT 2D islands seed is formed (Fig. 1b) consisting of the uniform thickness islands (more than 90% substrate area) with the atomically flat top surface (RMS roughness <50 pm), flat irregular form and the average lateral size from 100 nm to 250 nm.

At the second step, the deposition is stopped and the substrate is cooled down to 25 °С. Then, more silver is evaporated on the AFT 2D seed (Fig. 1c–e) until a continuous single-crystalline film is formed (Fig. 1f). At room temperature, the reduced surface diffusion length *D*^33^ and the hopping rate *ν* of adatoms arriving on the substrate lead to decreased upwards mass transport. On the other hand, Ag adatoms arriving on islands are hopping along the atomically flat top surfaces of the AFT 2D islands with almost no energy dissipation and easily get islands edges. Moreover, the strain relaxation results in the reduced step-edge *E*_*S*_ barrier for the adatoms on the islands surface^39,41^ and, thus, increased downward mass transport. Therefore, at the second step almost all the coming adatoms are adsorbed at the edges (perimeter) of the islands, spreading the dominant Ag(111) 2D islands and, eventually, coalescing the islands with each other, thus, completing the single-crystalline film. In the end, the strain that was accumulated in the first growing step relaxes primarily into interactions with the incoming adatoms improving the crystalline structure of the AFT 2D islands. Upon subsequent annealing at 320–480 °C, silver film crystalline structure and surface roughness are improved^42,43^, along with defect density reduction. In the next section, we demonstrate that SCULL process allow to overcome the well-known problem of the silver dewetting under elevated temperatures and deposit high quality sub-50 nm thick single-crystalline silver film.

## Results and Discussion

**Figure 2:**
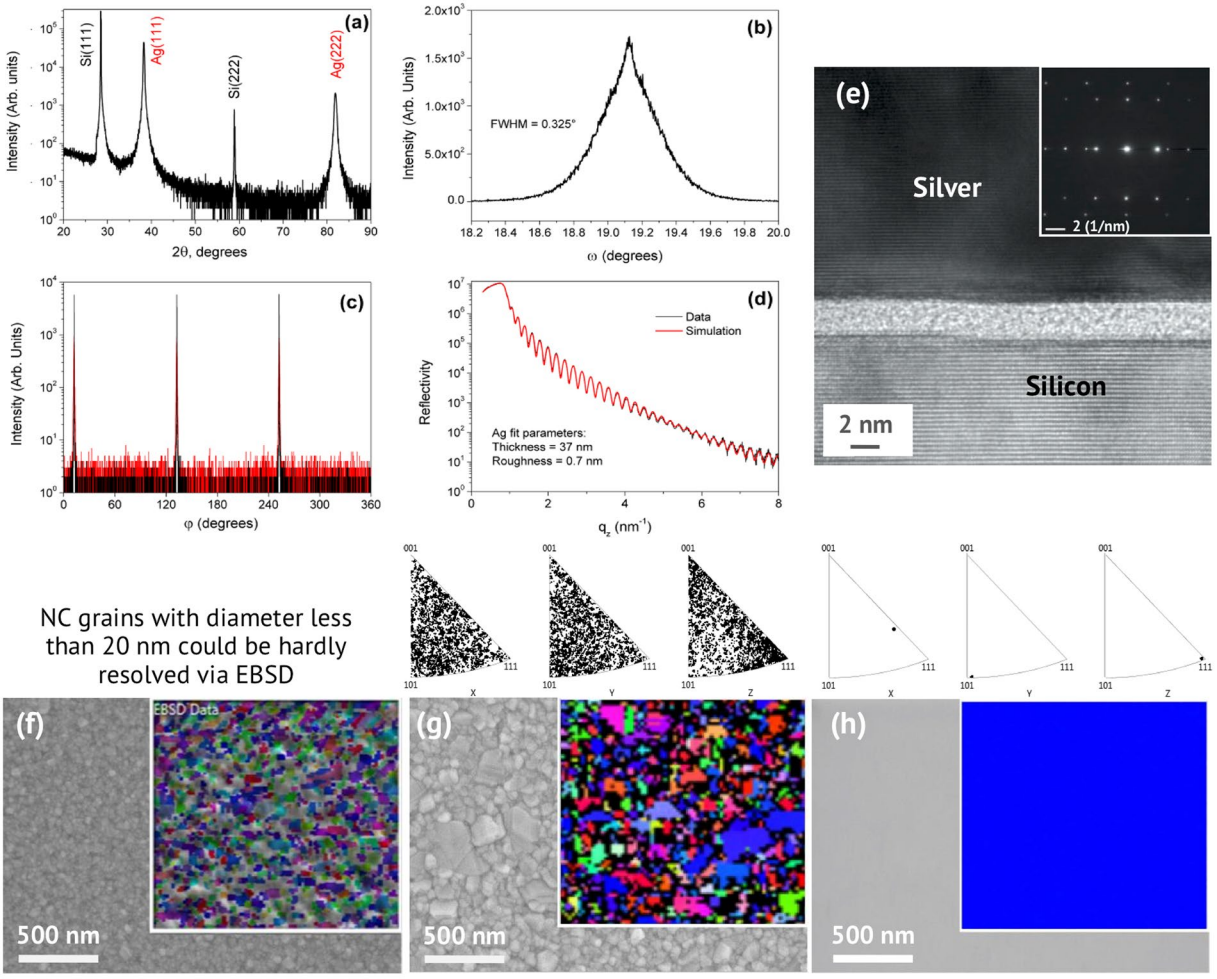
Microstructure characterization of a 37-nm-thick Si (111)/Ag (111) film (S1) and SEM images with EBSD insets of (NC), (PC) and (S1) films. (**a**) XRD (θ–2θ) pattern indicating only Ag (111) and Si (111) substrate peaks. (**b**) Measured transverse scan (rocking curve, ω-scan) through the Ag (111) diffraction peak. (**c**) Grazing incidence of the in-plane X-ray diffraction scan (phi-scans) of the Ag(111) plane. (**d**) X-ray reflectivity curve. (**e**) HRTEM image and the electron diffraction pattern (inset in the right corner), the growth direction is bottom-up. SEM images with EBSD insets of NC (**f**), PC (**g**) and S1 (**h**) silver films highlighting film grains. EBSD inverse pole figures are shown above the SEM images, demonstrating very tight crystal orientation density of the S1 film (**h**) along all the normal directions. Only a single domain is observed for S1 film in both small-scale 2 μm (**h**) and large-scale 400 μm scans (Fig. S6).

In this section, we demonstrate that grain boundaries, material purity (hence, grain boundaries purity), surface roughness (and associated surface chemical reactivity), and crystallinity imperfection contribute to optical properties of metal films in descending order of priority. We compare the results for six representative films: three SCULL single-crystalline films of 35 nm (S1), 70 nm (S4) and 100 nm (S5) nominal thickness, and three nominally 100-nm-thick polycrystalline films (PC, PCBG^31^, NC) with different grain size and purity (Table 1). Since dielectric permittivity is thickness independent^18,29^, optical properties of the Ag (111)/Si (111) films (S1, S4, S5) without grain boundaries and the same material purity properties were compared to estimate surface roughness and crystallinity impact. High-resolution wide-angle X-ray diffraction (XRD) rocking curves (Figs 2b, S4 and S5) with a full width at half maximum (FWHM) of 0.325°, 0.221°, 0.368° for the film thicknesses of 37 nm (S1), 68 nm (S4) and 99 nm (S5) indicate high quality film independent of its thickness, with minimal level of defects (see Supplementary Information for details). It is important to note that SCULL films deposited on a non lattice-matched Si (100) and Si (110) have predictably worse crystallinity, but also demonstrate atomically smooth surfaces with RMS roughness less that 4 Å (Table 1). High-resolution transmission electron microscopy (HRTEM) image (Fig. 2e) demonstrates the single-crystalline nature of the S1 silver film. Electron backscatter diffraction (EBSD) is used to analyze the domain structures and extract the average grain size (Table 1) of single-crystalline (Fig. 2h) and polycrystalline films (Figs 2f,g, S8c).

**Figure 3:**
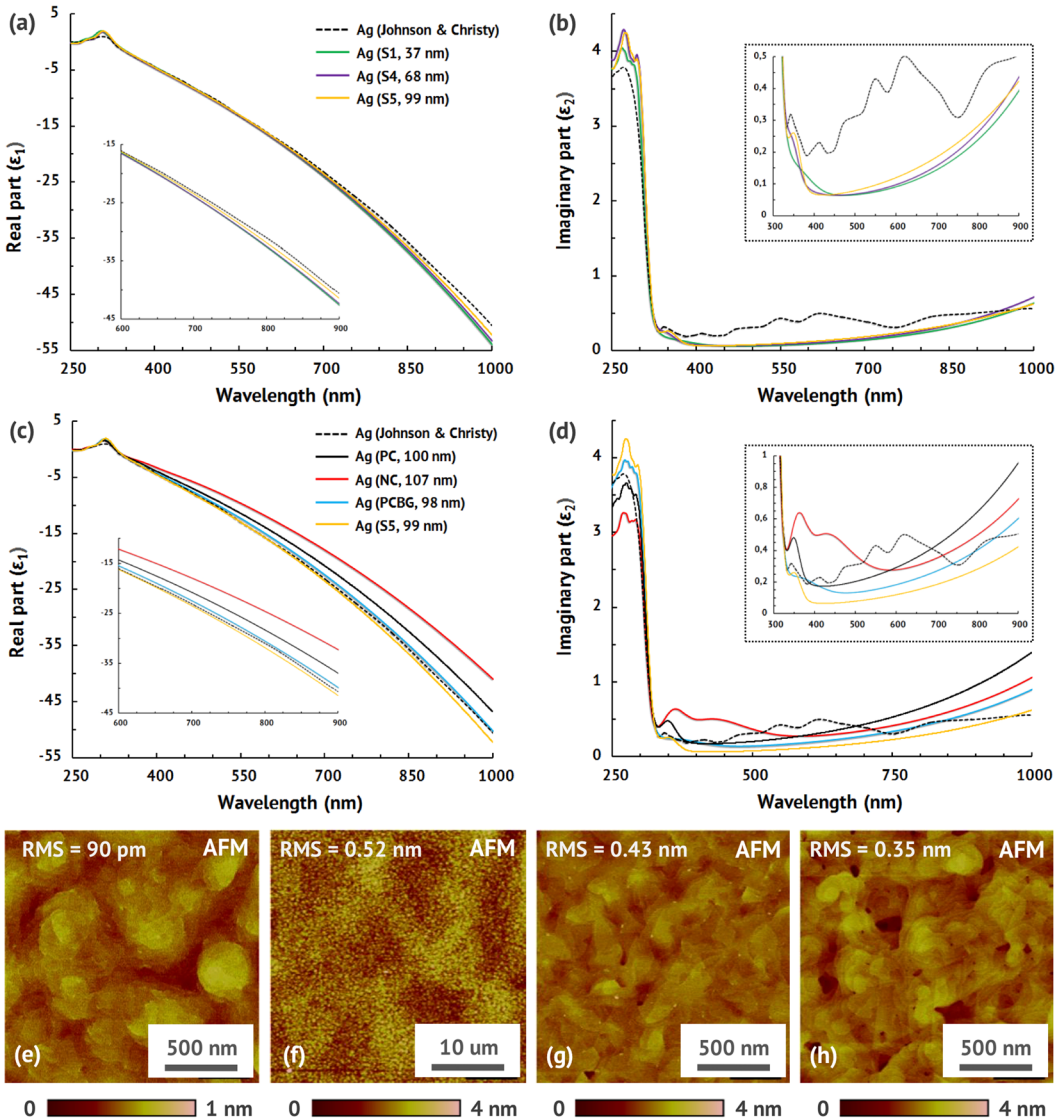
Optical properties and surface characterization. Real (**a**) and imaginary (**b**) part of the dielectric permittivity of the single-crystalline films (S1, S4, S5). Dielectric permittivity (**c**,**d**) of nominally 100-nm-thick single-crystalline (S5) and polycrystalline (PC, NC, PCBG) films. AFM scans of S1 (**e**), S4 (**g**) and M1 (**h**) films measured over a 2.5 × 2.5 μm^2^ area, and S1 (**f**) film, measured over a 50 × 50 μm^2^ area. All the films surfaces are continuous without pinholes and we observe no grain boundaries for single-crystalline films (**e**–**h**). The S1 film is extremely smooth with an atomic level of root mean square (RMS) roughness equal to 90 pm (**e**), which is the smoothest reported single-crystalline silver film. The RMS roughnesses of thicker films S4 and M1 are slightly larger, but still extremely smooth of 0.43 nm (**с**) and 0.35 nm (**d**).

To estimate losses and rank the films parameters contribution to optical properties a multi-angle spectroscopic ellipsometry is used (see Supplementary Information for details). We focus on the most practically useful NIR and visible wavelength region for silver lays above the interband transitions (λ > 325 nm), where the contribution to ε_1_ mainly comes from Drude terms (dc conductivity), but ε_2_ is defined by both intraband and interband components. We observe the dominating contribution of grain boundaries to dielectric permittivity (Fig. 3a–d), the real part becomes more negative with the increasing grains size (Fig. 3c) indicating higher conductivity. The NC film with a great number of small grains has the worst ε_1_ even compared to PC film deposited in a poor vacuum. In contrast, all the single-crystalline films have larger negative ε_1_ compared to JC and polycrystalline films. Observed decrease in negative ε_1_ (conductivity) is primarily due to increased number of structural defects (including grain boundaries) in the films leading to the increased electron-phonon interactions, which make the films less metallic. In general the same influence of the films grain size on ε_2_ is observed (Fig. 3d), except the PC film in 600–1000 nm wavelength range, which has larger losses than NC in spite of a bigger grain size. Indeed, it can be explained by poor PC film purity, which leads to increased Drude term of the imaginary part of the dielectric permittivity^44^, elevating losses at longer wavelengths (λ > 500 nm).

Material purity and surface roughness are the factors of the second priority in terms of silver dielectric permittivity in the 600–1000 nm and 325–600 nm wavelengths respectively. To demonstrate material purity effect, we compare the dielectric permittivity of relatively clean (NC, PCBG) and contaminated (PC) polycrystalline films with JC data. JC data^29^ was acquired from the thin films deposited near 170 times faster than the PC film (at very high evaporation rate of 60 Å s^−1^), leading to much purer silver film. Our measurements (Fig. 3c,d) indeed show larger negative ε_1_ and lower ε_2_ of JC data compared to all the polycrystalline films in the 600–1000 nm wavelengths. However, the above JC permittivity supremacy is almost neglected compared to the PCBG film, because of larger grains, which, in contrast, improving the film optical quality. These material purity dependencies can be attributed to an increase in the electron-phonon interaction as described above.

At the 325–600 nm wavelengths, with increasing surface roughness (averaged value) and surface morphology singularities (absolute number of surface nonuniformities) the ε_2_ is dramatically increased, and for the NC film it becomes more than five times and more than twice larger (Fig. 3d) compared to the S5 and PCBG films respectively. Furthermore, there are typical peaks in ε_2_ between 340 nm and 400 nm wavelengths for all the samples, and it is important to note that the S1 film peak amplitude is four times lower than the NC film peak amplitude. These ε_2_ spectrum features can be explained by internal interfaces effects^45^, that is, with the surface roughness and morphology increase the silver surface oxidation and the chemical reactivity is boosting. The observed typical peaks in ε_2_ are primarily due to the surface reaction with adsorbed sulphur^32,45^, which transforms the silver into a non-metal silver-sulphide (by transfer of S-ions through the interface). In case of polycrystalline films the surface topography (active surface area) plays the key role in the increased silver surface chemical reactivity leading to ε_2_ spectrum degradation close to interband transition threshold. For the single-crystalline films with the improving surface roughness (to sub-100 pm level) the typical peak associated with the interband transitions is almost eliminated (but is still present) due to better surface thermodynamic stability^32^ and weaker sorptivity to chemical elements from ambient.

In addition, SCULL silver films could be of a great interest for rapidly growing field of quantum plasmonics as atomically smooth single-crystalline films with low optical absorption and high conductivity can result in enhanced SPP propagation length. A theoretically predicted SPP propagation length for silver over two hundred microns^33^ and exceptional performance of plasmonic devices^9,31^ are experimentally demonstrated using SCULL silver films. We believe that it is the result of simultaneous synergistic effect from SCULL films dedicated single-crystalline nature, angstrom-scale surface roughness, process-induced high purity and thermodynamic stability^32^.

In conclusion, we have developed the two-step approach for e-beam evaporation of continuous atomically smooth single-crystalline metal films in a wide range of thicknesses down to 35 nm. The process provides thermodynamic, i.e. macroscopic, control of the film growth kinetics at atomic level and based on 2D crystal seed growth, known for 3D bulk materials, but not previously reported for thin films. The key feature of our approach is the combination of two mixed evaporation modes together with AFT 2D crystal seed growth self-controlled by quantum size effects, which enables deposition of perfect single-crystalline metal films on non-ideally lattice-matched substrates, even with imperfect standard PVD tools and typical process deviations. We have demonstrated 35–100 nm thick single-crystalline silver films with the sub-100 pm surface roughness, perfect crystallinity (XRD rocking curves through Ag(111) peaks have FWHM of 0.2–0.4°), much lower optical losses compared to JC data^29^ and SPP propagation length above two hundred microns^33^. Using SCULL silver films as golden samples we have demonstrated that grain boundaries, material purity, surface roughness and microstructure imperfection contribute to optical properties in descending order of priority. The proposed process has been approved for silver, gold and aluminum single-crystalline films growth on silicon, sapphire and mica substrates. We believe that SCULL process could be used for deposition of various atomically smooth single-crystalline metal thin films, as well, it could be easily integrated in planar top-down device fabrication technology. The unique physical and optical properties of SCULL films may open fundamentally new possibilities in nanophotonics^1,15^, biotechnology^6,10^ and quantum technologies^9^.

## Methods

### Preparation of epitaxial films

Silver thin films were deposited on prime-grade degenerately doped Si(111), Si(100), Si(110) wafers (0.0015–0.005 Ω-cm) and muscovite mica substrates using 10 kW e-beam evaporator (Angstrom Engineering) with a base pressure lower than 3 × 10^−8^ Torr. We first cleaned the wafers in a 2:1 sulfuric acid: hydrogen peroxide solution (80 °C), followed by further cleaning in isopropanol to eliminate organics. Finally, we placed the wafers in 49% hydrofluoric acid for approximately 20 s to remove the native oxide layer. After oxide removal, we immediately transferred the wafers into the evaporation tool and pumped the system down to limit native oxide growth. Mica substrates were cleaved perpendicular to the c-axis to reveal fresh surfaces, prior to deposition. All films were grown using 5N (99.999%) pure silver. Films were deposited with rate of 0.5–10 Å·s^−1^ measured with quartz monitor at approximate source to substrate distance of 30 cm. Deposition is done in two steps using SCULL process.

### X-ray diffraction (XRD)

X-ray diffraction was studied by means of the Rigaku SmartLab diffractometer. To study texture and azimuthal orientation of silver crystals in relation to silicon monocrystal axes, φ-scans for both Ag and Si layers were measured from 0° to 360° with 0.052° step. Rocking curves (or ω-scans, 0.001° step) were applied to characterize in-plane perfection of silver crystals. In each sample, observed reflections were caused by sets of crystallographic planes with divisible Miller indices. This means that crystallographic planes of silver crystals were parallel to planes of the substrate with the same Miller indices. Ag(111) was parallel to Si(111), Ag(110)//Si(110) and Ag(100)//Si(100). A difference in value of lattice parameter of silver on substrate with various Si orientations, if any, was lower than uncertainty of the method of investigation. Averaged out of all samples lattice parameter was 4.076 ± 0.004 Å, which was in good agreement with known value for pure Ag atomic weight^7^. Curves of φ-scans consisted of sharp reflections for all the samples. The number and position of these reflections coincided with standard (111), (110) and (001) FCC crystal projections. Also, the position of reflections from the silver film matched in each case to a position of those of a silicon substrate. This result meant that each studied silver film possessed biaxial texture. Analysis of 2θ/ω and φ-scans showed that each studied sample was biaxially textured silver film on mono-crystalline silicon substrate with the following epitaxial relationships: (111)Ag//(111)Si:[111]Ag//[111]Si, (110)Ag//(110)Si:[110]Ag//[110]Si and (001)Ag//(001)Si:[001]Ag//[001]Si. Additionally, φ-scans showed that misorientation of these films and a substrate did not exceed 0.1°. Appearance of a Si(222) forbidden reflection is in accordance with recently published investigations. Since full-width-at-half-maximum (FWHM) of a rocking curve profile serves as a practical numerical characteristic of mosaic spread in thin crystalline films for plasmonics, precise determination of this value is of importance.

### Atomic force microscopy (AFM)

The atomic force microscope Bruker Dimension Icon with SCANASYST-AIR-HR probe (with nominal tip radius of 2 nm) was used. All AFM images were obtained by using PeakForce Tapping mode with ScanAsyst imaging and the scanned area was 2.5 × 2.5 μm^2^ and 50 × 50 μm^2^. Nanoscope software was utilized to analyze the images and extract root mean square roughness.

### Ellipsometry

Dielectric functions of the silver films were measured using a multi-angle spectroscopic ellipsometer (SER 800, Sentech GmbH). Additionally, ellipsometers in three different laboratories have been crosschecked (for single-crystalline silver films on Si(111)) to eliminate the possibility of systematic errors. We specifically measured the optical constants of the HF treated silicon substrate used in the deposition process to eliminate any discrepancy and uncertainty introduced by the substrate. These measured silicon optical constants and silver thickness are fixed in the subsequent data fitting for all samples, and only the silver parameters are allowed to change.

Modeling and analysis were performed with the ellipsometer SENresearch 4.0 software. The models were developed in cooperation with Sentech GmbH application department. Measurement spectral wavelength range was from 240 to 1000 nm, with an interval of approx. 2 nm, and the reflected light was analyzed at incidence angles of 50°, 60°, 70°. To characterize the optical losses, the real (ε_1_) and imaginary (ε_2_) parts of the dielectric permittivity were extracted by fitting the measured raw ellipsometric data (Ψ and Δ). In our fitting, we used a bilayer Ag/Si structural model and a simple phenomenological Brendel-Bormann (BB) oscillator model to interpret both the free electron and the interband parts of the dielectric response of our samples:

1 $$\hat{\varepsilon }(\omega )={\varepsilon }_{\infty }-\frac{{\omega }_{p}^{2}}{{\omega }^{2}+i{{\rm{\Gamma }}}_{{\rm{D}}}{\rm{\omega }}}+{\sum }_{j=1}^{k}{\chi }_{j}(\omega ),$$

where ω_p_ is the plasma frequency, ${\varepsilon }_{\infty }$ is the background dielectric constant, ${{\rm{\Gamma }}}_{{\rm{D}}}$ is Drude damping, ${\chi }_{j}(\omega )$ is BB oscillators interband part of dielectric function, and k is the number of BB oscillators used to interpret the interband part of the spectrum.

Modern ellipsometers are capable of obtaining a presice optical data. Using flexible and sophisticated models and analysis software, it is possible accurately determine the optical constants of materials. While data obtaining creates no difficulties for high quality samples, analysis is not trivial. Extracting reliable permittivity is challenging because it is an inverse problem. Polarization ratio of reflected light are measured and the optical constants of the structure under investigation and layer thicknesses are retrieved.

The mean square error (MSE) is a crucial parameter to quantify the quality of fitted permittivity parameters. However, a small MSE alone is not a conclusive proof that the model is totally reliable. A model contains highly correlated parameters, so it is possible to have multiple solutions with similarly low MSE values. A strong correlation exist between thickness and optical constants in absorptive metal thin films and lead to unreliable permittivity values. To verify that the final fit solution is truly unique, we need to do a test showing that there is indeed a best fit at a singular value of a chosen parameter. The parameter we chose to perform the uniqueness test on is the independelty-measured thickness of the film. By fixing the thickness of the film at a measured value with tolerance 2 nm, while letting the other parameters vary during the fitting process, we calculated the MSE of each final fit result. For all samples, the mean square errors, representing the quality of the match between the measured and theoretically calculated dielectric functions, were the best for measured thickness less than 1.3°. From these uniqueness tests, we conclude that our model is indeed reliable and the retrieved optical constants are valid.

### Profilometry

The stylus profiler KLA Tencor P17 (with Durasharp 38-nm tip radius stylus) was used. All measurements were done by using 0.5 mg taping strength, scan rate was 2 μm·s^−1^ and the scanned line length was 20 μm.

### Scanning electron microscopy

In order to check the quality and uniformity of the deposited layers silver films surfaces after deposition were investigated by means of a scanning electron microscope Zeiss Merlin with a Gemini II column. All SEM images were obtained using in-lens detector and the accelerating voltage 5 kV and working distance from the sample to detector from 1 to 4 mm. Magnifications 3000, 7000, 15000 and 50000 were used to fully analyze samples.

### Electron back scattered diffraction (EBSD) characterisation

The Ag films were observed and structurally characterized by field emission scanning electron microscopy (FE-SEM: Zeiss Merlin Gemini II). The crystal orientation maps of the Ag films were obtained by FE-SEM equipped with an EBSD system (NordlysNano from Oxford Instrument, Oxford Instruments Corp., UK). EBSD patterns were acquired at the following shooting modes: tilt angle – 70°, accelerating voltage – 10 keV, probe current – 1.7 nA and scan sizes 2 × 2 μm^2^ and 20 × 20 μm^2^ for SC film. EBSD has proven to be a useful tool for characterizing the crystallographic orientation aspects of microstructures at length scales ranging from dozens of nanometers to millimeters in the scanning electron microscope. Detector provides single grains detection by means of orientation measurement based on acquired Kikuchi patterns. Colored image represents grains orientation map, correlation between colors and orientations is shown on triangle diagram at the bottom left corner. We extract average grain size for our polycrystalline films using the embedded software package for an EBSD image processing AZtecHKL software package.

We extract average grain size for our polycrystalline films using the embedded software package for an EBSD image processing. The NC film was e-beam evaporated onto a liquid nitrogen cooled quartz substrate, the conditions were adjusted so that the film had an average grains size around 20 nm.

### Transmission electron microscopy (TEM)

In order to check the quality and crystallinity of the nominally 35-nm deposited silver film on Si(111) its crossection made by ion milling was investigated by means of a transmission electron microscope TITAN 300. TEM image was obtained using in-lens detector and the accelerating voltage 100 kV, spot size 3.

## Supplementary information

Supplementary Information
