# Supplementary material for: Quantum Engineering of Atomically Smooth Single-Crystalline Silver Films
Source: Sci Rep. 2019 Aug 22;9:12232. doi: 10.1038/s41598-019-48508-3 (PMC6706392; doi:10.1038/s41598-019-48508-3)
Supplement: Supplementary file 1 — Supplementary Information [file 41598_2019_48508_MOESM1_ESM.pdf]

Supplementary Information

Quantum Engineering of Atomically Smooth Single-Crystalline Silver Films

Ilya A. Rodionov[*1,2], *Aleksandr S. Baburin*[1,2], *Aidar R. Gabidullin*[1,2], *Sergey S. Maklakov*[4], *Sven Peters*[3], *Ilya A. Ryzhikov*[1,4], *and Alexander V. Andriyash*[2]

[1]FMN Laboratory, Bauman Moscow State Technical University, Moscow, Russian Federation

[2]Dukhov Research Institute of Automatics, Moscow, Russian Federation,

[3]SENTECH Instruments GmbH, Berlin, Germany,

[4]Institute for Theoretical and Applied Electromagnetics RAS, Moscow, Russian Federation

*e-mail: irodionov@bmstu.ru

Table of content

1. **Results and discussion**
2. **Methods**
   2.1. Deposition
   2.2. X-Ray Diffraction (XRD)
   2.3. Atomic Force Microscopy (AFM)
   2.4. Ellipsometry
   2.5. Thickness measurement
   2.6. Profilometry
   2.7. Scanning electron microscopy (SEM)
   2.8. Electron back scattered diffraction (EBSD) characterisation
   2.9. Transmission electron microscopy (TEM)
3. **SCULL technique for Au and Al thin films deposition**
4. **Supplementary Information Figures**

1. Results and discussion

Grain boundaries, material purity (hence, grain boundaries purity), surface roughness (and associated surface chemical reactivity), and crystalline imperfection contribute to optical properties of metallic films in descending order of priority. To demonstrate this, we compare the results for six representative films: three SCULL single-crystalline films of 35 nm (S1), 70 nm (S4) and 100 nm (S5) nominal thickness, and three nominally 100-nm-thick polycrystalline films (PC, PCBG, NC) with different grain size and purity. To estimate grain boundaries and surface roughness impact, we compare nominally 100-nm-thick polycrystalline films (PC, PCBG, NC) with various grain size and identical surface roughness (Table 1) with the single-crystalline (S5) film. The NC film was e-beam evaporated onto a liquid nitrogen cooled quartz substrate, the conditions were adjusted so that the film had an average grains size around 20 nm. The PCBG film was e-beam evaporated using optimized two-step process[6] to obtain an average grains size lager than 500 nm. In order to highlight material purity effect and emulate highly cited JC silver films, which was obtained under poor vacuum conditions ($4 \cdot 10^{-6}$ Torr, oil-pumped), a 100-nm-thick polycrystalline film (PC) was deposited under 10^{-6} Torr preasure (dry-pumped). Since dielectric permittivity is thickness independent[11,13], optical properties of the Ag(111)/Si(111) films (S1, S4, S5) without grain boundaries and the same material purity properties were compared to estimate a surface roughness and crystallinity impact. High-resolution wide-angle X-ray diffraction (XRD) rocking curves (see the XRD section for details) with a full width at half maximum (FWHM) of 0.325 °, 0.221°, 0.368 ° for the film thicknesses 37 nm (S1), 68 nm (S4) and 99 nm (S5) indicate thickness independent film high quality with minimal level of defects, which is comparable to previously best reported even thicker single-crystalline silver PVD films[18]. It is important to note the SCULL films deposited on a non lattice-matched Si (100) and Si (110) have predictably worse crystallinity, but also demonstrate atomically smooth surfaces with RMS roughness less that 4 Å (Table 1). The high-resolution transmission electron microscopy (HRTEM) image (Fig. 9) demonstrates the single-crystalline nature of the S1 silver film. Electron backscatter diffraction (EBSD) is used to analyse the domain structures and extract average grain size (Table 1) of single-crystalline (Fig. 7c, 8d, 8e) and polycrystalline films (Fig. 7a, 7b, 8a, 8b, 8c). Only a single domain is observed in both small-scale 2 μm (Fig. 8d) and large-scale 400 μm scans (Fig. 8f).

To estimate losses and rank the films parameters contribution to optical properties a multi-angle spectroscopic ellipsometry is used (see the Ellipsometry section for details). We focus on the most practically useful NIR and visible wavelength region for silver lays above the interband transitions ($\lambda > 325$ nm), where the contribution to ε_1 mainly comes from Drude terms (dc conductivity), but ε_2 is defined by both intraband and interband components. We observe the dominating contribution of grain boundaries to dielectric permittivity (Fig. 10), the real part

becomes more negative with increasing grains size (Fig. 10c) indicating higher conductivity. The NC film with a great number of small grains has the worst ε_1 even compared to PC film deposited in a poor vacuum. Opposite, all the single-crystalline films have larger negative ε_1 compared to JC and polycrystalline films. The observed decrease in negative ε_1 (conductivity) is primarily due to increased number of structural defects (including grain boundaries) in the films leading in the the increased electron-phonon interactions, which make the films less metallic. In general the same influence of the films grain size on ε_2 is observed (Fig. 10d), except the PC film in 600-1000 nm wavelength range, which has lager losses than NC in spite of bigger grain size. Indeed, it can be explained by poor PC film purity, which leads to increased Drude term of the imaginary part of the dielectric permittivity[14], elevating losses at longer wavelengths ($\lambda > 500$ nm).

Material purity and surface roughness are the factors of the second priority in term of silver dielectric permittivity in the 600-1000 nm and 325-600 nm wavelengths respectively. To demonstrate material purity effect, we compare the dielectric permittivity of relatively clean (NC, PCBG) and duty (PC) polycrystalline films with widely cited JC data. One should note that JC dielectric permittivity was averaged from the measurements of the films with 30.4 nm and 37.5 nm thickness deposited at very high evaporation rate (60 $Å\cdot s^{-1}$). A dielectric permittity is thickness independent, however, the JC films were deposited near 170 times faster than the PC film (see the Deposition section), leading to much more pure silver film. Our measurements (Fig. 10c, 10d) indeed show larger negative ε_1 and lower ε_2 of JC data compared to all the polycrystalline films in the 600-1000 nm wavelengths. However, the above JC permittivity supremacy is almost neglected compared to the PCBG film, because of the very big grains, which, in contrast, improving the film optical quality. These material purity dependencies can be attributed to an increase in the electron-phonon interaction as described above.

The surface morphology of the films is characterized by AFM. All the films surfaces are continuous without pinholes and we observe no grain boundaries for single-crystalline films (Fig. 9). The S1 film is extremely smooth with an atomical level of root mean square (RMS) roughness equal to 90 pm (Fig. 9a), which is the smoothest reported single-crystalline silver film. The RMS roughness of thicker films S4 and M1 are slightly larger, but still extremely smooth of 0.43 nm (Fig. 9b) and 0.35 nm (Fig. 9c). At the 325-600 nm wavelengths, with increasing surface roughness (averaged value) and surface morphology singularites (absolute number of surface nonuniformities) the ε_2 is dramatically increased, and for the NC film it becomes more than five times and more than twice larger (Fig. 10d) compared to the S5 and PCBG films respectively. Futhermore, there are typical peaks in ε_2 between 340 nm and 400 nm wavelengths for all the samples, and it is important to note that the S1 film peak amplitude is four times lower than the NC film peak amplitude. These ε_2 spectrum features can be explained by internal interfaces effects[14], that is, with the surface

roughness and morphology increase a silver surface oxidation and chemical reactivity is boosting. The observed typical peaks in ε_2 are primarily due to the surface reaction with adsorbed sulphur[15,16] which transforms, by transfer of S-ions through the interface, the silver into a non-metal silver-sulphide. In case of polycrystalline films the surface topography (active suface area) plays the key role in the increased silver surface chemical reactivity leading to ε_2 spectrum degradation close to interband transition threshold. For the single-crystalline films with improving the surface roughness (to sub-100-pm level) the typical peak associated with the interband transitions is almost eliminated (but is still present) due to silver surface perfect thermodynamic stability and weaker sorbtivity to chemical elements from ambient.

As atomically smooth single-crystalline silver films with extremely low optical absorption and high conductivity can result in an enhanced SPP propagation length, SCULL films should be of a great interest for high performance plasmonic applications. The extremely low losses and improved plasmonic properties of SCULL films was confirmed by demonstrating SPP propagation length above two hundred microns over the 100 nm single-crystalline silver film[17], which is twice longer than previously reported experimental results[10,11,18]. We believe that it is the result of the SCULL films unique characteristics and synergistic effect from the dedicated single-crystalline nature, atomically smooth surface, process-induced high purity and thermodynamic stability.

In this paper, we have demonstrates the two-step PVD SCULL process to obtain a continuous atomically smooth single-crystalline metallic films deposition over a wide range of thicknesses. The fundamental idea of the process involves quantum engineering of an effective underlying layer (1st step), which thermodinamically emulate a lattice-matched substrate for the targeted metal, followed by a single-crystalline film growth (2nd step) on the just syntesied lattice-matched substrate. The process provides the single-crystalline metallic films growth on non-ideally lattice-matched substrates without underlayers using a high vacuum electron-beam evaporator. The extremely low optical losses and improved plasmonic perfomance have been confirmed by demonstrating the longest reported SPP propagation length above two hundred microns[8].

2. Methods

2.1 Deposition

Silver thin films were deposited on prime-grade degenerately doped Si(111), Si(100), Si(110) wafers (0.0015-0.005 $\Omega\cdot$cm) and muscovite mica substrates using 10 kW e-beam evaporator (Angstrom Engineering) with a base pressure lower than $3\cdot10^{-8}$ Torr. We first cleaned the wafers in a 2:1 sulfuric acid: hydrogen peroxide solution (80˚C), followed by further cleaning in isopropanol to eliminate organics. Finally, we placed the wafers in 49% hydrofluoric acid for approximately 20 s to remove the native oxide layer. After oxide removal, we immediately transferred the wafers into

the evaporation tool and pumped the system down to limit native oxide growth. Mica substrates were cleaved perpendicular to the c-axis to reveal fresh surfaces, prior to deposition. All films were grown using 5N (99.999%) pure silver. Films were deposited with rate of 0.5-10 $Å \cdot s^{-1}$ measured with quartz monitor at approximate source to substrate distance of 30 cm. Deposition is done in two steps using SCULL process. First, seed island layer is deposited on the elevated temperature substrate. At the second step, evaporation is stopped and the substrate is cooled to room temperature. Then, silver deposition is started on seed layer until continuous single-crystalline film is formed. Different temperature in a range from room temperature to 450˚C were used.

2.2 X-Ray Diffraction (XRD)

X-ray diffraction was studied by means of the Rigaku SmartLab diffractometer . PaTo study texture and azimuthal orientation of silver crystals in relation to silicon monocrystal axes, φ-scans for both Ag and Si layers were measured from 0° to 360° with 0.052° step. Rocking curves (or ω-scans, 0.001° step) were applied to characterize in-plane perfection of silver crystals. In each sample, observed reflections were caused by sets of crystallographic planes with divisible Miller indices. This means that crystallographic planes of silver crystals were parallel to planes of the substrate with the same Miller indices. Ag(111) was parallel to Si(111), Ag(110)//Si(110) and Ag(100)//Si(100). A difference in value of lattice parameter of silver on substrate with various Si orientations, if any, was lower than uncertainty of the method of investigation. Averaged out of all samples lattice parameter was 4.076 ± 0.004 Å, which was in good agreement with known value for pure Ag atomic weight[7]. Curves of φ-scans consisted of sharp reflections for all the samples (Fig. 1-5). The number and position of these reflections coincided with standard (111), (110) and (001) FCC crystal projections. Also, the position of reflections from the silver film matched in each case to a position of those of a silicon substrate. This result meant that each studied silver film possessed biaxial texture. Analysis of 2θ/ω and φ-scans showed that each studied sample was biaxially textured silver film on mono-crystalline silicon substrate with the following epitaxial relationships: (111)Ag//(111)Si:[111]Ag//[111]Si, (110)Ag//(110)Si:[110]Ag//[110]Si and (001)Ag//(001)Si:[001]Ag//[001]Si. Additionally, φ-scans showed that misorientation of these films and a substrate did not exceed 0.1°. Appearance of a Si(222) forbidden reflection is in accordance with recently published investigations[8]. Since full-width-at-half-maximum (FWHM) of a rocking curve profile serves as a practical numerical characteristic of mosaic spread[1] in thin crystalline films for plasmonics[9], precise determination of this value is of importance. The values derived were: 0.325 for S1 sample, 0.829 for S2 and 0.831 for S3. The FWHM value for S1 sample was comparable to that of a previously reported single-crystalline films for modern plasmonic applications but of larger thicknesses: 0.116° for 1-μm thick Ag film[10] and 0.22° for 200-nm thick

Ag film[11]. S7 sample showed same diffractometric character as S1 and a rocking curve FWHM value of 0.368°.

2.3. Atomic Force Microscopy (AFM)

The atomic force microscope Bruker Dimension Icon with SCANASYST-AIR-HR probe (with nominal tip radius of 2 nm) was used. All AFM images were obtained by using PeakForce Tapping mode with ScanAsyst imaging and the scanned area was 2.5×2.5 μm^2 and 50×50 μm^2. Nanoscope software was utilized to analyze the images and extract root mean square roughness.

2.4. Ellipsometry

Dielectric functions of the silver films were measured (Table 2) using a multi-angle spectroscopic ellipsometer (SER 800, Sentech GmbH). Additionally, ellipsometers in three different laboratories have been crosschecked (for single-crystalline silver films on Si(111)) to eliminate the possibility of systematic errors. We specifically measured the optical constants of the HF treated silicon substrate used in the deposition process to eliminate any discrepancy and uncertainty introduced by the substrate. These measured silicon optical constants and silver thickness are fixed in the subsequent data fitting for all samples, and only the silver parameters are allowed to change.
Modeling and analysis were performed with the ellipsometer SENresearch 4.0 software. The models were developed in cooperation with Sentech GmbH application department. Measurement spectral wavelength range was from 240 to 1000 nm, with an interval of approx. 2 nm, and the reflected light was analyzed at incidence angles of 50˚, 60˚, 70˚. To characterize the optical losses, the real (ε_1) and imaginary (ε_2) parts of the dielectric permittivity were extracted by fitting the measured raw ellipsometric data (Ψ and Δ). In our fitting, we used a bilayer Ag/Si structural model and a simple phenomenological Brendel-Bormann (BB) oscillator model[12] to interpret both the free electron and the interband parts of the dielectric response of our samples:

$$\hat{\varepsilon}(\omega) = \varepsilon_\infty - \frac{\omega_p^2}{\omega^2 + i\Gamma_D\omega} + \sum_{j=1}^{k} \chi_j(\omega), \quad (1)$$

where ω_p is the plasma frequency, ε_∞ is the background dielectric constant, Γ_D is Drude damping, $\chi_j(\omega)$ is BB oscillators interband part of dielectric function, and k is the number of BB oscillators used to interpret the interband part of the spectrum.

Modern ellipsometers are capable of obtaining a presice optical data. Using flexible and sophisticated models and analysis software, it is possible accurately determine the optical constants of materials. While data obtaining creates no difficulties for high quality samples, analysis is not trivial. Extracting reliable permittivity is challenging because it is an inverse problem. Polarization

ratio of reflected light are measured and the optical constants of the structure under investigation and layer thicknesses are retrieved.

The mean square error (MSE) is a crucial parameter to quantify the quality of fitted permittivity parameters. However, a small MSE alone is not a conclusive proof that the model is totally reliable. A model contains highly correlated parameters, so it is possible to have multiple solutions with similarly low MSE values. A strong correlation exist between thickness and optical constants in absorptive metal thin films and lead to unreliable permittivity values. To verify that the final fit solution is truly unique, we need to do a test showing that there is indeed a best fit at a singular value of a chosen parameter. The parameter we chose to perform the uniqueness test on is the independelty-measured thickness of the film. By fixing the thickness of the film at a measured value with tolerance 2 nm, while letting the other parameters vary during the fitting process, we calculated the MSE of each final fit result. For all samples, the mean square errors, representing the quality of the match between the measured and theoretically calculated dielectric functions, were the best for measured thickness less than 1.3°(Table 1. S1-5). From these uniqueness tests, we conclude that our model is indeed reliable and the retrieved optical constants are valid.

2.5. Thickness measurement

The film thickness was measured independently by SEM crossection, profilometer. Zeiss Merlin with a Gemini II column was used for measurement, obtained image resolution is better than 1 nm. Fabricated film step was measured by stylus profiler KLA Tencor P17 (with Durasharp 38-nm tip radius stylus), repetability of vertical size measurement by profilometer is 0.4 nm. The measurments for nominally 65-nm thick film (S4) are presented on Fig. 6.

2.6. Profilometry

The stylus profiler KLA Tencor P17 (with Durasharp 38-nm tip radius stylus) was used. All measurements were done by using 0.5 mg taping strength, scan rate was 2 $\mu m \cdot s^{-1}$ and the scanned line length was 20 μm.

2.7. Scanning electron microscopy

In order to check the quality and uniformity of the deposited layers silver films surfaces after deposition were investigated by means of a scanning electron microscope Zeiss Merlin with a Gemini II column. All SEM images were obtained using in-lens detector and the accelerating voltage 5 kV and working distance from the sample to detector from 1 to 4 mm. Magnifications 3000, 7000, 15000 and 50000 were used to fully analyze samples.

2.8. Electron back scattered diffraction (EBSD) characterisation

The Ag films were observed and structurally characterized by field emission scanning electron microscopy (FE-SEM: Zeiss Merlin Gemini II). The crystal orientation maps of the Ag films were obtained by FE-SEM equipped with an EBSD system (NordlysNano from Oxford Instrument, Oxford Instruments Corp., UK). EBSD patterns were acquired at the following shooting modes: tilt angle – 70˚, accelerating voltage – 10 keV, probe current – 1.7 nA and scan sizes 2x2 um^2 and 20 x 20 um^2 for SC film. EBSD has proven to be a useful tool for characterizing the crystallographic orientation aspects of microstructures at length scales ranging from dozens of nanometers to millimeters in the scanning electron microscope. Detector provides single grains detection by means of orientation measurement based on acquired Kikuchi patterns. Colored image represents grains orientation map, correlation between colors and orientations is shown on triangle diagram at the bottom left corner. We extract average grain size for our polycrystalline films using the embedded software package for an EBSD image processing AZtecHKL software package .

The growth direction is bottom-up along the [111] direction. EBSD images of NC (Fig. 7a), PC (Fig. 7b) PCBG(Fig. 7c) and S1 (Fig. 7d,e) silver films demonstrating single-crystalline nature without grain boundaries.

We extract average grain size for our polycrystalline films using the embedded software package for an EBSD image processing. The NC film was e-beam evaporated onto a liquid nitrogen cooled quartz substrate, the conditions were adjusted so that the film had an average grains size around 20 nm. The PCBG film (Fig. 7c) was e-beam evaporated using optimized two-step process[31] to obtain an average grains size lager than 500 nm. Only a single domain is observed in both small-scale 2 μm (Fig. 7d) and large-scale 400 μm scans (Fig. 7e), confirming the quality of our single-crystalline silver films.

2.9. Transmission electron microscopy (TEM)

In order to check the quality and crystallinity of the nominally 35-nm deposited silver film on Si(111) its crossection made by ion milling was investigated by means of a transmission electron microscope TITAN³ 300. TEM image (Fig. 11) was obtained using in-lens detector and the accelerating voltage 100 kV, spot size 3.

2.9. Surface plasmon-polariton (SPP) propagation measurement[17]

Measurement of the SPP propagation length is a rather complicated experimental problem and usually consists of two steps: (1) effective excitation of SPP waves and (2) measurement of the SPPs propagation length. One of the most accessible, convincing and informative is the method of measuring the SPP propagation length based on registration of SPP waves which are excited by

laser radiation. The SPPs are excited by using a nanoslits array patterned on a metal film surface. Registration of the launched SPP is performed through the scattering the SPP on the nanogrooves (detectors of SPP waves) located on the metal film surface on the SPP propagation way Fig. 12(c). We call this method “the far-field optical microscopy of SPP waves”. In this method, the excitation of SPP is realized through the scattering of laser radiation on the array of nanoslits and the subsequent scattering of the launched SPP on nanogrooves. Detection of scattered radiation on nanogrooves in far field helps to visualize SPP propagation as well as to measure the SPP propagation length. The measurements were made with silver films both on transparent, and on opaque substrates. We note that optical microscopy of SPP waves on metallic films made on an opaque substrate is much more complicated in implementation, in comparison with the case of transparent substrates. This complexity is caused by the fact that excitation of the SPPs and their detection are carried out from the same side of the substrate. In this case, the same microscope objective is used to excite SPP and then to detect radiation scattered by the SPP on nanostructures - detectors. The main problem here is to detect a weak scattering signal of SPP on the nanostructures - detectors against a giant reflected and scattered signals from the laser used to excite SPP. Two types of nanostructures were created on the silver film surface using focused-ion-beam milling. One of them was designed to excite SPP wave and was formed by a matrix of 20 slits nanostructures with a nanoslits spacing of $\Lambda1$= 780 nm and a slit size of 120 nm × 20 μm. Another one served as a detector of the SPP wave, and was formed by a matrix of 15 nanogrooves with a 40 nm depth, 120 nm width and 40 μm length with a distance between nanogrooves equal to 20 μm. Figure 12(a,b) shows the electron microscope images of the created nanostructures. Figure 12(c) shows the scheme for measuring the SPP propagation length. The measurements were performed using an inverted Nikon Eclipse/Ti-U microscope. To excite SPP wave, we used a CW semiconductor laser with a tunable wavelength around 780 nm, having ultra narrow linewidth. This laser light was focused with a microscope objective (x20) into a 6 μm spot on the array of nanoslits. The period of nanoslits array $\Lambda1$ was chosen to effectively excite SPP on the film surface at orthogonal incidence of the laser beam on the sample. The period was determined from the relation $Re(k_{SPP}) \approx G + k_0 \sin \alpha$, where k_{SPP} is the wave number of the SPP waves, $G = 2\pi/\Lambda1$ is the modulus of the reciprocal lattice vector of the nanoslits array, α is the angle of laser radiation incidence on the sample (α = 900), $k_0 = \omega/c$ is the wavenumber of laser light. We fabricated series of parallel nanogrooves on the way of SPP propagation. The radiation scattered on these nanogrooves is recorded with the same microscope objective lens on a 2D CCD camera (Princeton Instrument, PhotonMax). The radiation is proportional to the intensity of the SPP waves at the location of the nanogroove, and the detection of the scattered light from the array of nanogrooves allows us to measure a change of the SPP intensity during its propagation. To increase the contrast of the measured signal, we used the fact

that the radiation formed due to scattering of SPP on nanogrooves has different polarization compared to incident laser light. We used two crossed polarizers having a residual extinction ratio of 1:1000. One of them was installed in the laser beam. Another one was installed just before CCD camera in such a way to block most of laser light reflected from the sample. Such arrangement of the polarizers helped us to substantially reduce laser radiation reflected and scattered from the sample. Figure 13(a) shows the Ag film surface optical image with fabricated nanostructures which were fabricated using focus-ion-beam milling method. The figure clearly shows both types of nanostructures. One is the array of nanoslits, designed to excite the SPP and which is visible as a rectangle. The distance between nanoslits is too small to be resolved by optical microscope. The figure also clearly shows distinctly separated nanogrooves. These nanogrooves are arranged parallel to the nanoslits and are on the way of the SPP propagation. Scattering of the SPP on each nanogroove allows us to visualize the SPP, and also to measure the SPP propagation length. Figure 3(b) shows the optical image of the silver film surface when it is irradiated by laser radiation at wavelength 780 nm. As it can be seen from the figure, the strong scattering of laser radiation on the array of nanoslits and the excitation of SPP on the Ag film surface appear. The SPP propagates and scatters on nanogrooves, giving possibility to visualize SPP propagation in optical microscope. Each of the spot on a nanogroove has elliptical shape. The smaller diameter d_1 of this spot is determined by a diffraction limit of used optical objective. The larger diameter of the spot d_2 is determined by width of SPP. Thus the optical image clearly shows the divergence of the SPP due to diffraction: as the distance from the excitation region of the SPP wave increases, the spot diameter d2 (from the scattering of the SPP wave by the nanogrooves) becomes higher. The spot size on a nanogroove located close to the array of nanoslit is $d_2(0\ \mu m) = 6\ \mu m$ and corresponds to the diameter of the exciting laser beam. On the nanogroove located at L = 275 μm from the first nanogroove, the corresponding spot size is much lager: $d_2(275\ \mu m) = 38\ \mu m$. As can be seen from the Fig. 13(b), the scattering signals from the nanogrooves are represented by the narrow peaks with the width equal to $d_1 = 2\ \mu m$, which is determined by the resolution of the objective lens with a numerical aperture NA = 0.4 ($d_1 \approx \lambda/N\ A$). It can be seen from Fig. 13(b) that the amplitudes of the scattering signal of SPP on the nangrooves are different and decrease with a distance between each nanogroove and the array of nanoslits because of the losses of the SPP in Ag thin film. The change in the amplitudes is well approximated by an exponential curve with a characteristic length equal to 93 μm. This measured SPP decay length is determined by three factors: (1) absorption losses of the SPP in the silver film, (2) losses of the SPP due to scattering on each nanogroove, and (3) divergence of the SPP waves caused by its diffraction. We will show below that from these measurements it is possible to extract the SPP propagation length determined only by the losses in the silver film. We measured losses of a SPP on each nanogroove, which equals to 4.8%. Briefly, to

measure the losses we have created another set of structures with a distance between nanogrooves two times lower compared to the structure presented on the Fig. 13. Thus the SPP crosses two times more nanogrooves during its propagation compared to the case presented on Fig. 13. The set of nanoslits and nanogrooves was created on the same Ag film with a distance about 0.5 mm from the structures presented on Fig. 13. For the measurements it is important that the nanogrooves are identical in the both structures. This was controlled by electron microscopy. To measure losses of SPP on nanogrooves we measured SPP decay curve for the both structures. The measured difference was attributed to losses of SPP on nanogrooves because number of nanogrooves is different and it permits to find losses of SPP on individual nanogroove. The SPP diffraction was taken into account by integrating the signal along each nanogroove, using the image of Fig. 13(b), which corresponds to scattering of the SPP by the nanogroove. Figure 14 shows the dependence of the SPP intensity attenuation, taking into account the losses at each nanogroove and the diffraction of the SPP wave. As can be seen from the figure, the data are well approximated by an exponential curve with an attenuation length characterizing SPP propagation length on a surface of Ag film $L_{SPP} = 194 \pm 23$ μm. In this figure, the zero position on the x axis corresponds to the edge of the nanoslits array (used to excite the SPP). It is also seen from the figure that even at a distance of 300 μm from the excitation region, the SPP still contains a significant amount of energy to be easily detected.

Figure 15 shows the dependence of the SPP intensity attenuation for gold thin film, taking into account the losses at each nanogroove and the diffraction of the SPP wave. As can be seen from the figure, the data are well approximated by an exponential curve with an attenuation length characterizing SPP propagation length on a surface of Au film $L_{SPP} = 53$ μm. In this figure, the zero position on the x axis corresponds to the edge of the nanoslits array (used to excite the SPP).

3. SCULL technique for Au and Al thin films deposition

The proposed SCULL technique was also used for gold and aluminum films deposition. It allowed to obtain ultra-smooth single-crystal films of these metals (Fig. 16).

For both gold and aluminum film root mean square roughnesses of less than 1 nm and optical characteristics exceeding those reported in the literature were obtained.

4. Supplementary Information Figures.

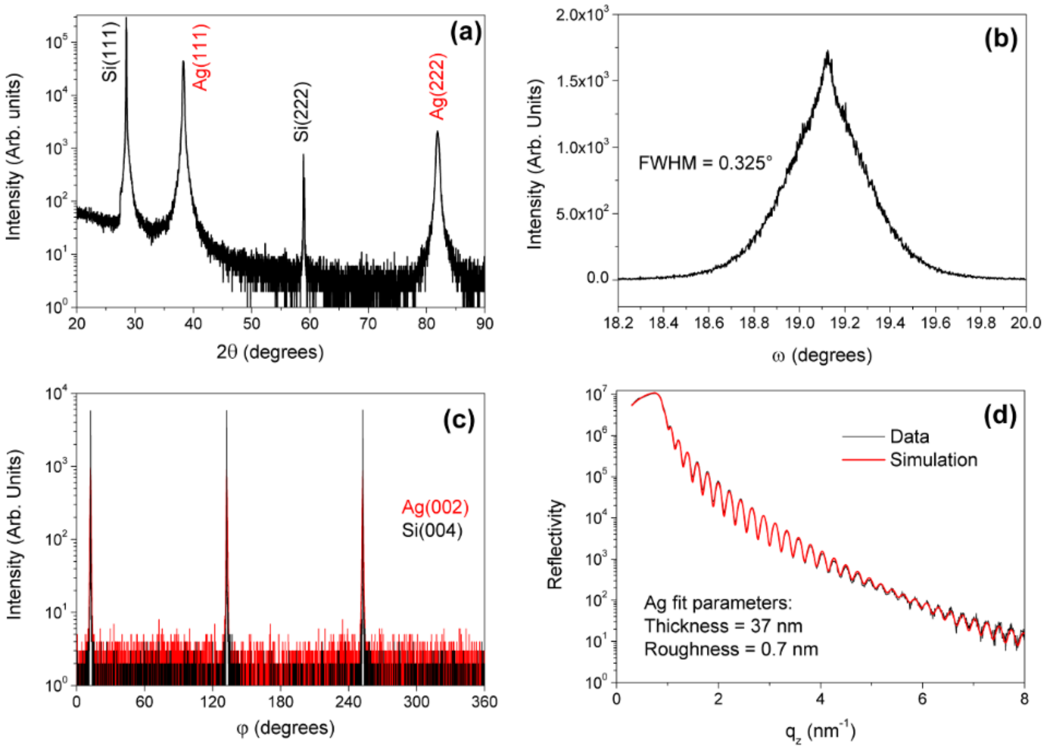

Supplementary Figure 1. | XRD characterization of a nominally 35-nm-thick Si(111)/Ag(111) film (S1). (**a**) High-resolution X-ray diffraction (θ-2θ) pattern. (**b**) Measured transverse scan (rocking curve, ω-scan) through the Ag(111) diffraction peak. (**c**) Grazing incidence of the in-plane X-ray diffraction scan (phi-scans) of the Ag(111) plane. (**d**) X-ray reflectivity curve.

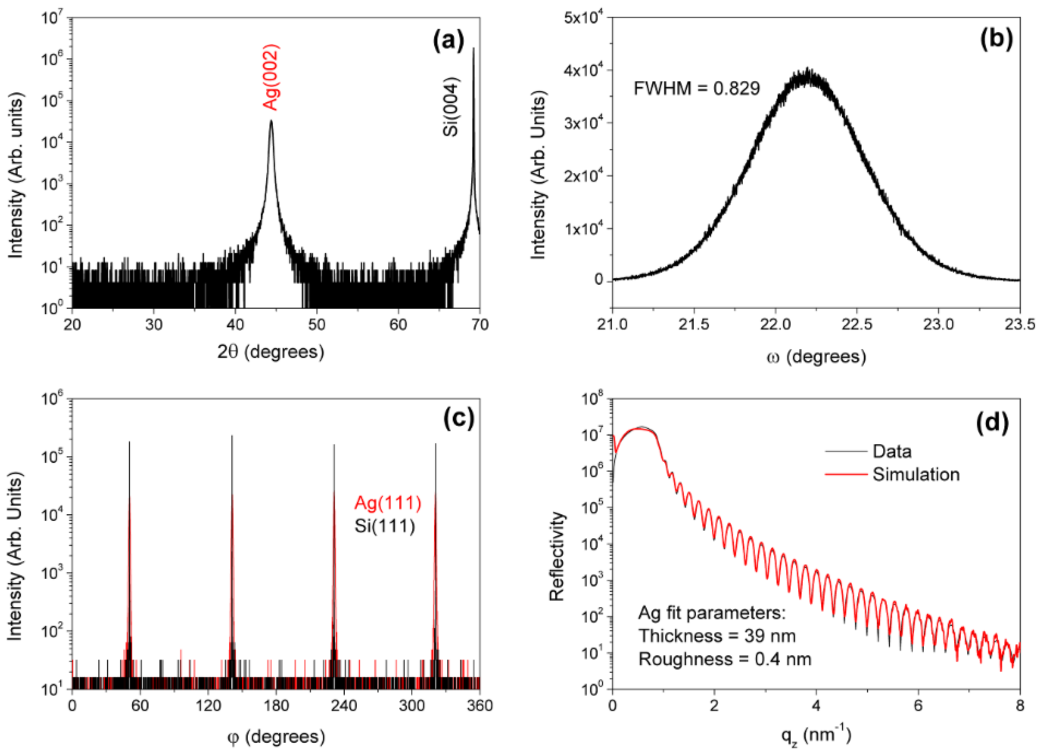

Supplementary Figure 2. | XRD characterization of a nominally 35-nm-thick Si(100)/Ag(100) film (S2). (**a**) High-resolution X-ray diffraction (θ-2θ) pattern. (**b**) Measured transverse scan (rocking curve, ω-scan) through the Ag(100) diffraction peak. (**c**) Grazing incidence of the in-plane X-ray diffraction scan (phi-scans) of the Ag(100) plane. (**d**) X-ray reflectivity curve.

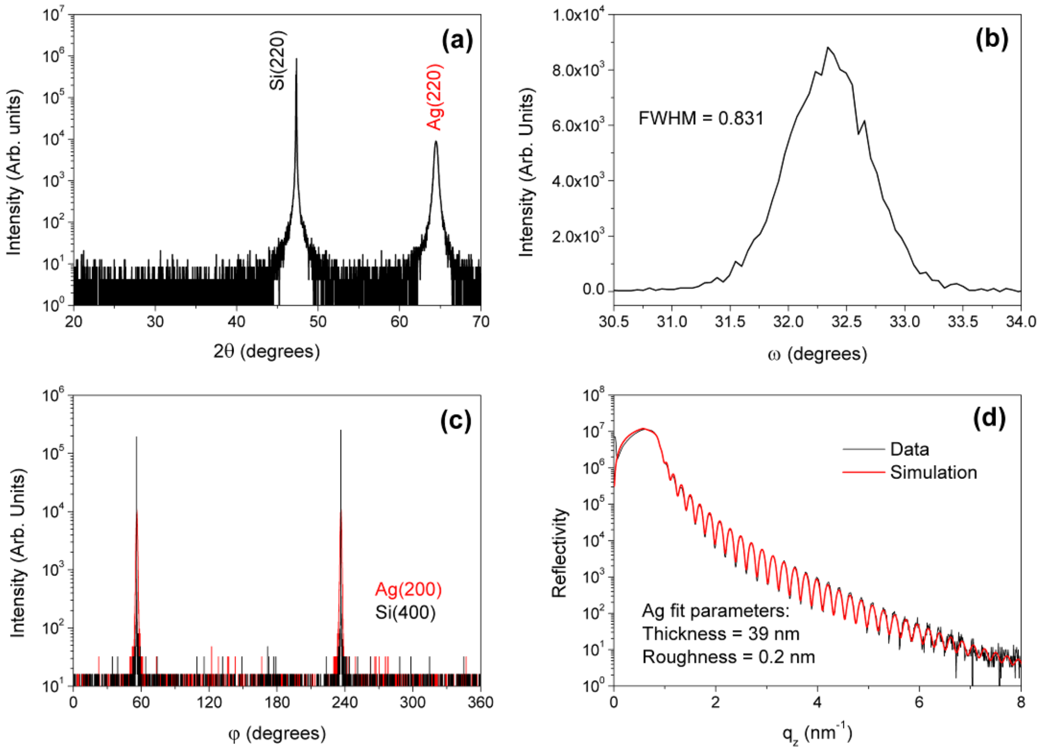

Supplementary Figure 3. | XRD characterization of a nominally 35-nm-thick Si(110)/Ag(110) film (S3). (**a**) High-resolution X-ray diffraction (θ-2θ) pattern. (**b**) Measured transverse scan (rocking curve, ω-scan) through the Ag(110) diffraction peak. (**c**) Grazing incidence of the in-plane X-ray diffraction scan (phi-scans) of the Ag(110) plane. (**d**) X-ray reflectivity curve.

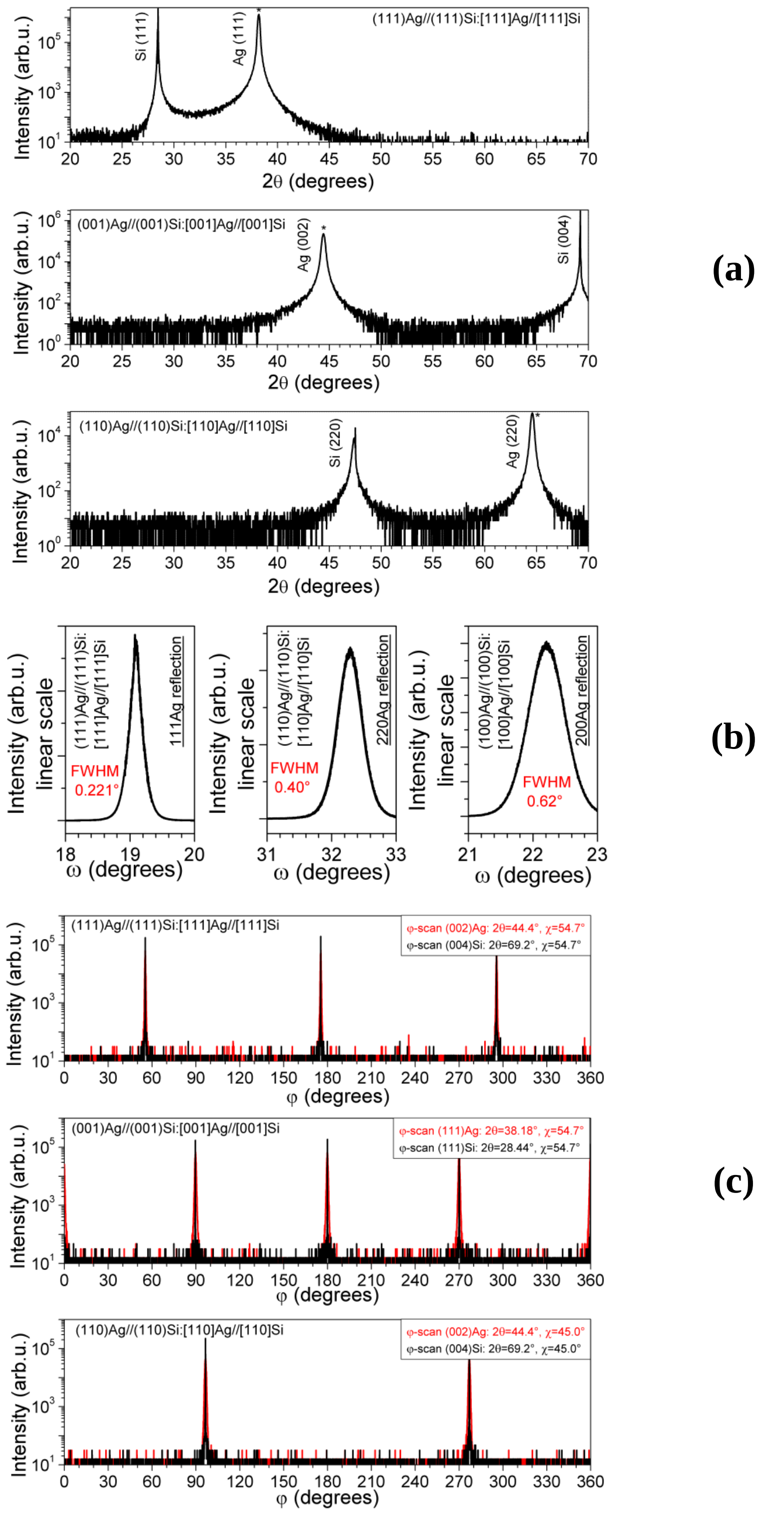

Supplementary Figure 4. | XRD characterization of a nominally 65-nm-thick Ag on Si(111), Si(100), Si(110) substrates respectively. (a) High-resolution X-ray diffraction (θ-2θ) pattern. (b) Measured transverse scan (rocking curve, ω-scan) through the Ag diffraction peak. (c) Grazing incidence of the in-plane X-ray diffraction scan (phi-scans) of the Ag plane.

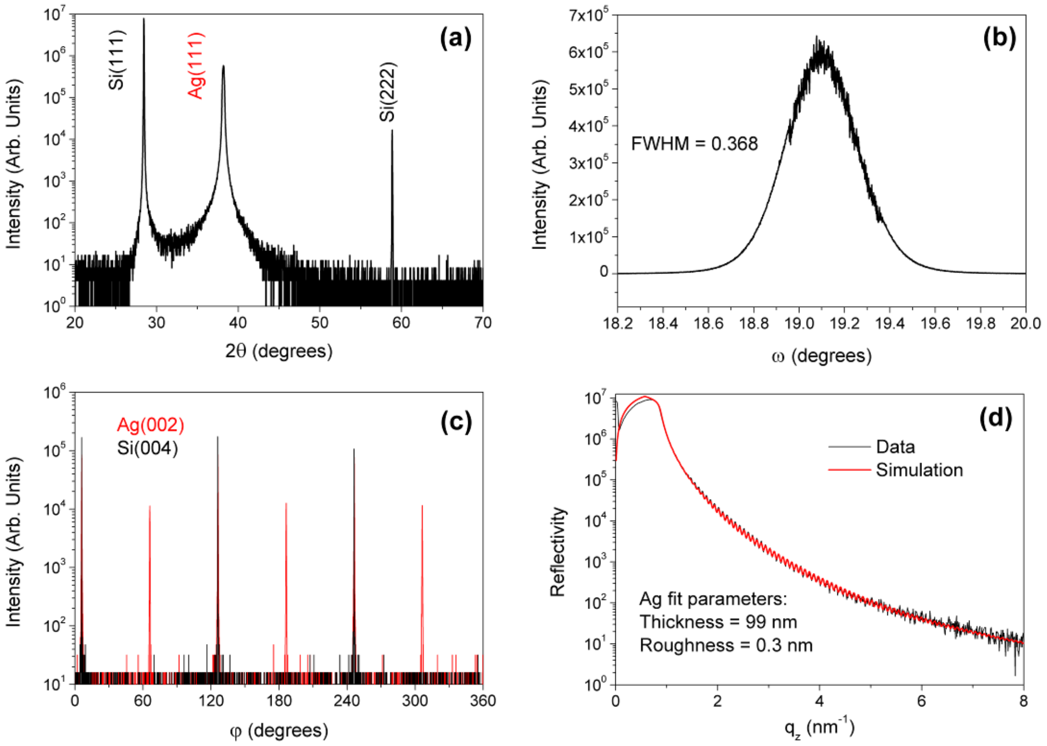

Supplementary Figure 5. | XRD characterization of a nominally 100-nm-thick Si(111)/Ag(111) film (S5). (**a**) High-resolution X-ray diffraction (θ-2θ) pattern. (**b**) Measured transverse scan (rocking curve, ω-scan) through the Ag(111) diffraction peak. (**c**) Grazing incidence of the in-plane X-ray diffraction scan (phi-scans) of the Ag(111) plane. (**d**) X-ray reflectivity curve.

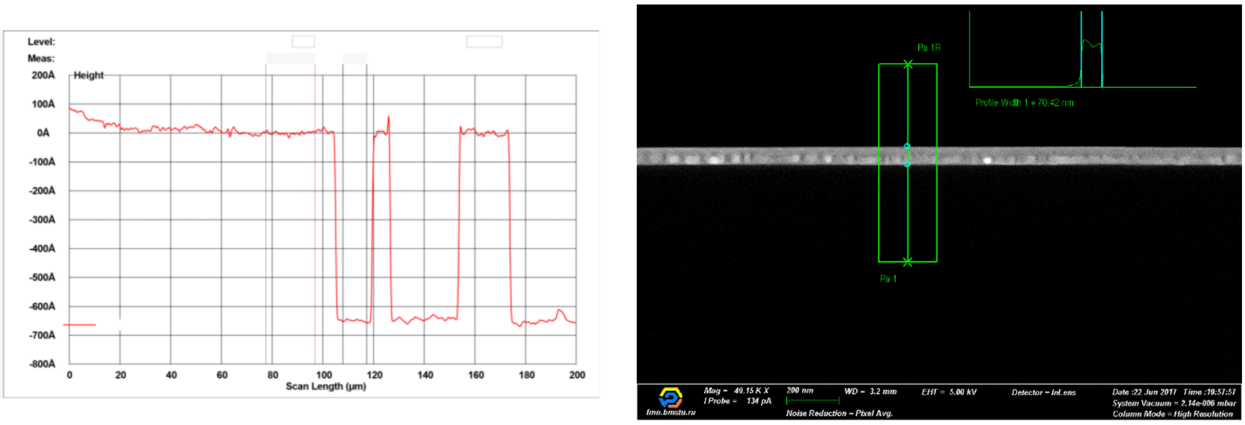

Supplementary Figure 6. | Thickness measurments of a nominally 70-nm-thick Si(111)/Ag(111) film (S4). (**a**) Profilometer step height measurment (**b**) SEM cross-section.

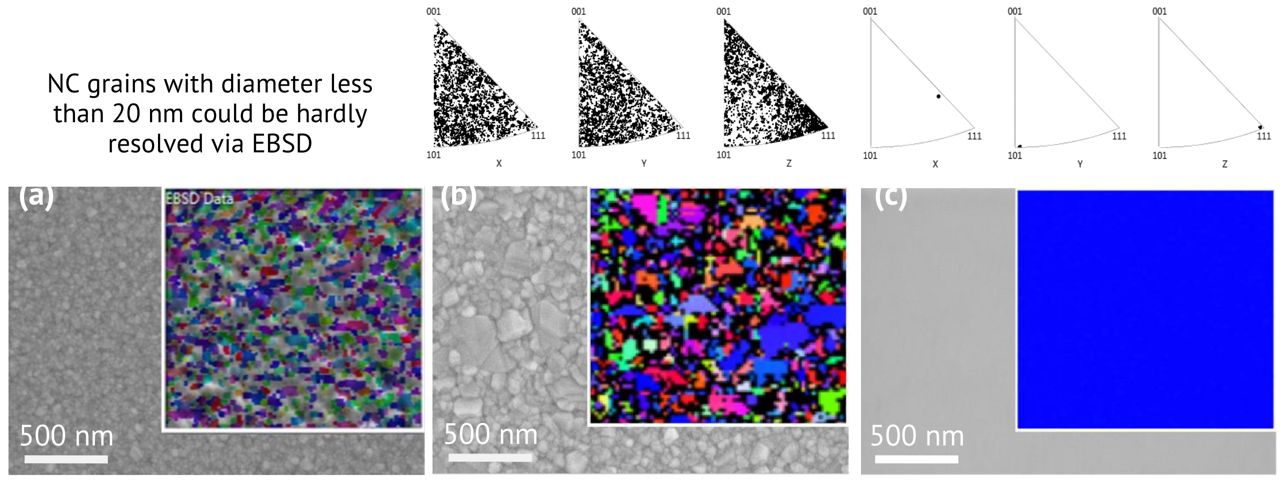

Supplementary Figure 7. | SEM images with EBSD insets. NC (**a**), PC (**b**) and S1 (**c**) silver films highlighting film grains. EBSD inverse pole figures are shown above the SEM images, demostrating very tight crystal orientation density of the S1 film (c) along all the normal directions.

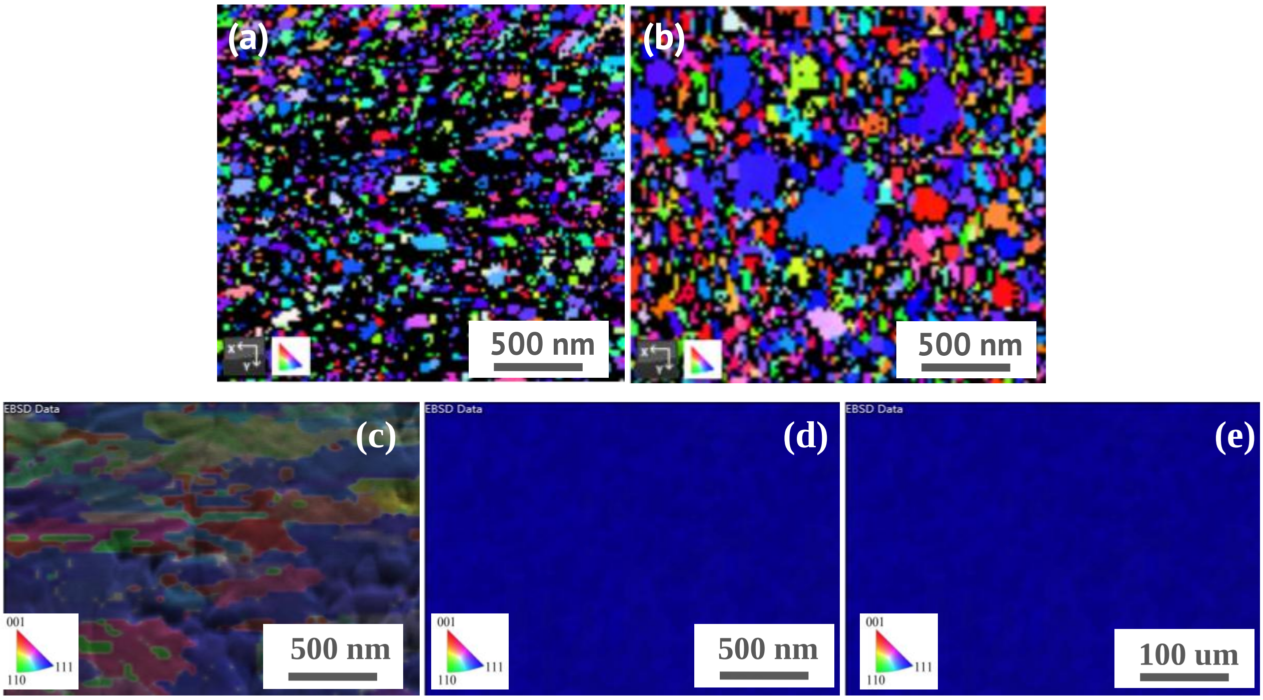

Supplementary Figure 8. | EBSD images of silver thin films. Nanocrystalline NC (a), polycrystalline PC (b), polycrystalline PCBG (c) and single-crystalline S1 (d, e) silver films. Only a single domain is observed in both small-scale 2 μm (d) and large-scale 400 μm scans (e), confirming the high quality and single-crystalline nature without grain boundaries over a large length scale.

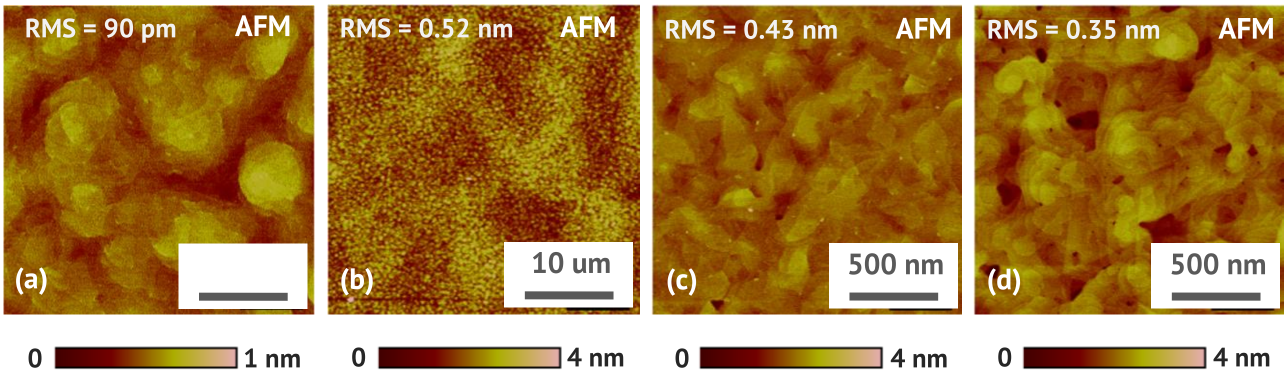

Supplementary Figure 9. | AFM scans. S1 (**a**), S4 (**b**) and M1 (**c**) films measured over a 2.5×2.5 μm^2 area, and S1 (**d**) film, measured over a 50×50 μm^2 area.

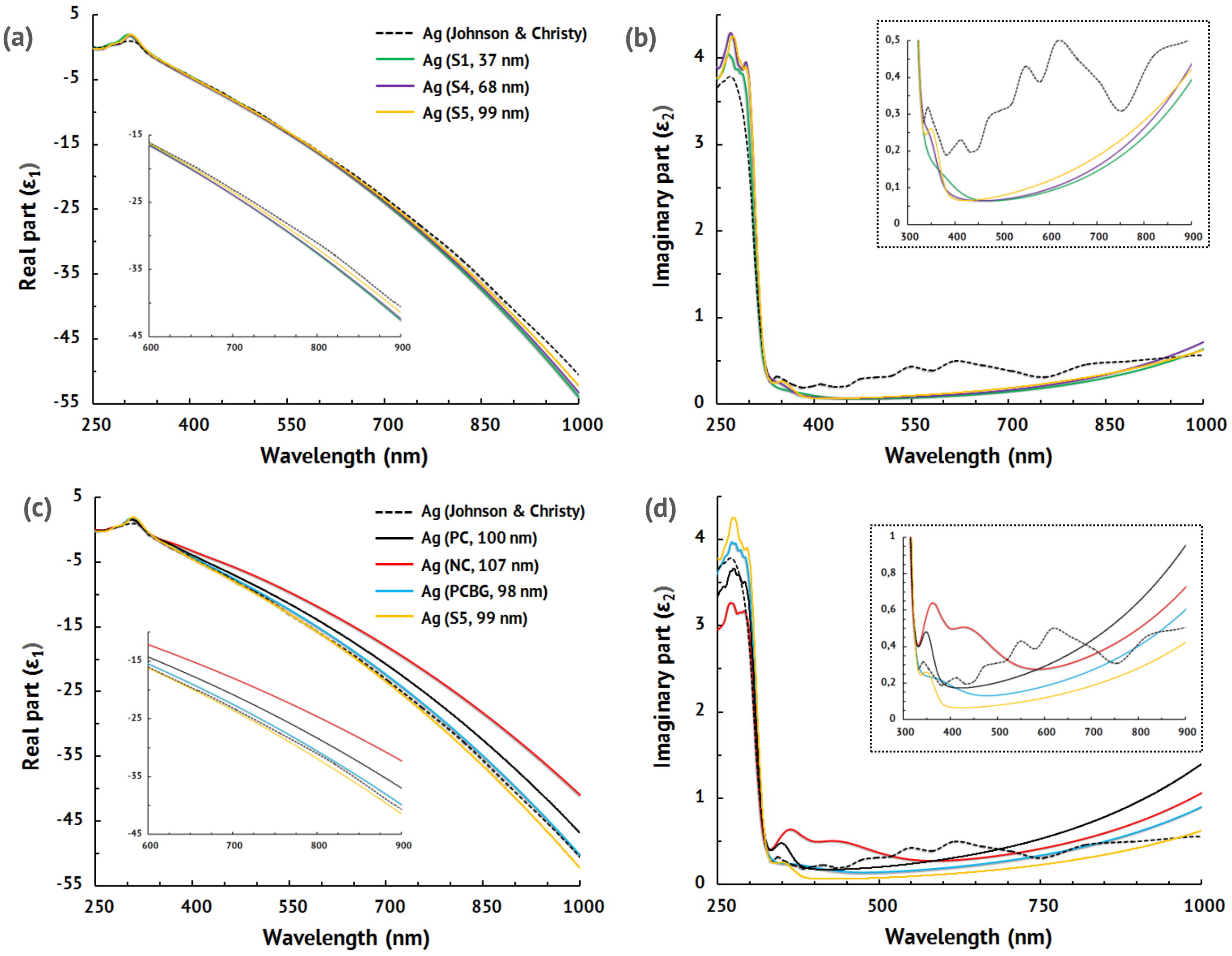

Supplementary Figure 10. | Silver films dielectric permittivity. Real (**a**) and imaginary (**b**) part of the dielectric permittivity of the single-crystalline films (S1, S4, S5). Dielectric permittivity (**c, d**) of nominally 100-nm-thick single-crystalline (S5) and polycrystalline (PC, NC, PCBG) films.

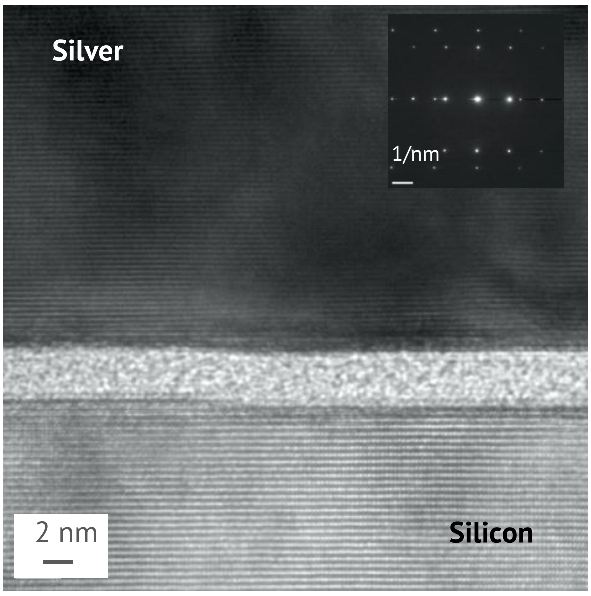

Supplementary Figure 11. | HRTEM image and the electron diffraction pattern (inset in the right corner). The growth direction is bottom-up.

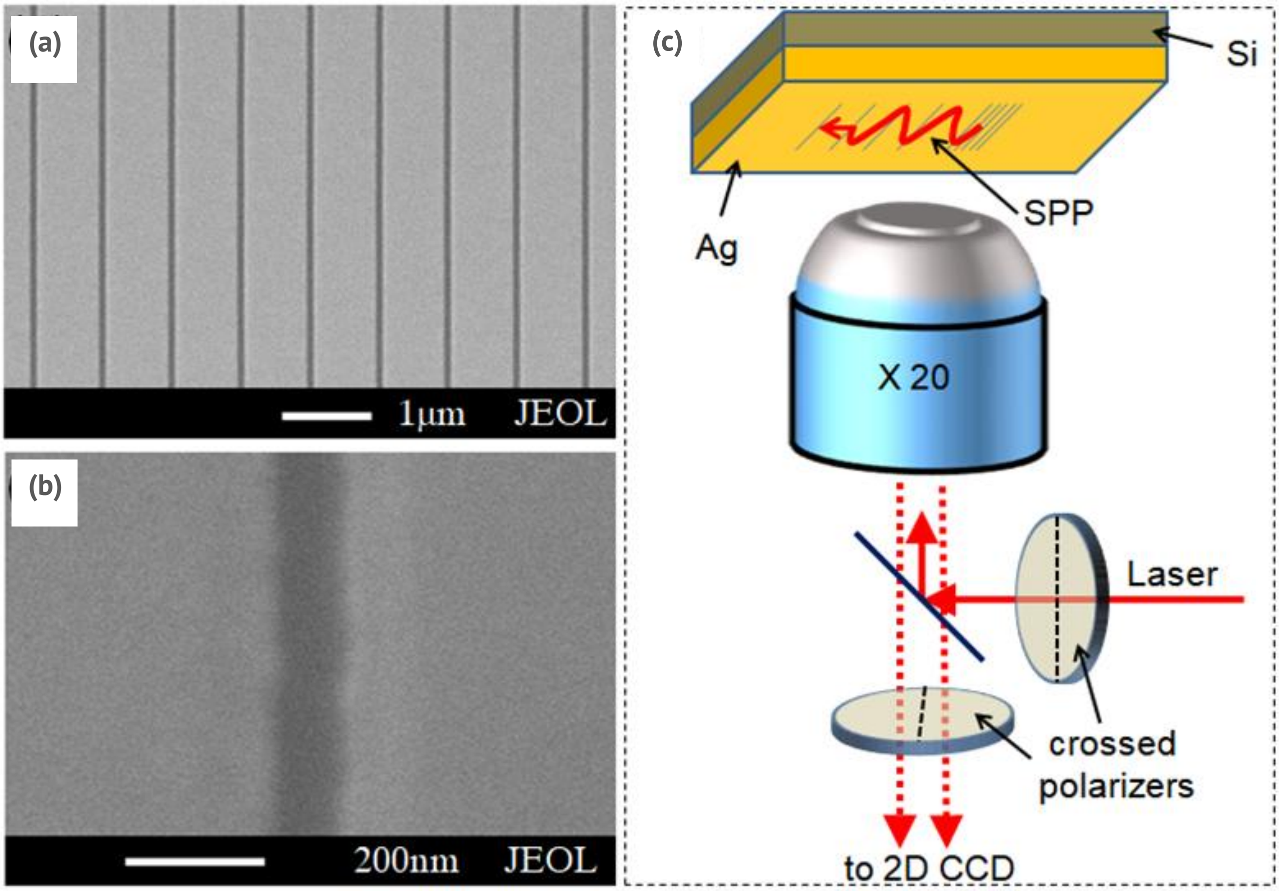

Supplementary Figure 12. | Electron microscopy of SPP[17]: (a) an electron microscope image of nanoslits array on the Ag film used to excite SPP, (b) an electron microscope image of a nanogroove used to detect SPP, (c) a schematic diagram of the experimental setup.

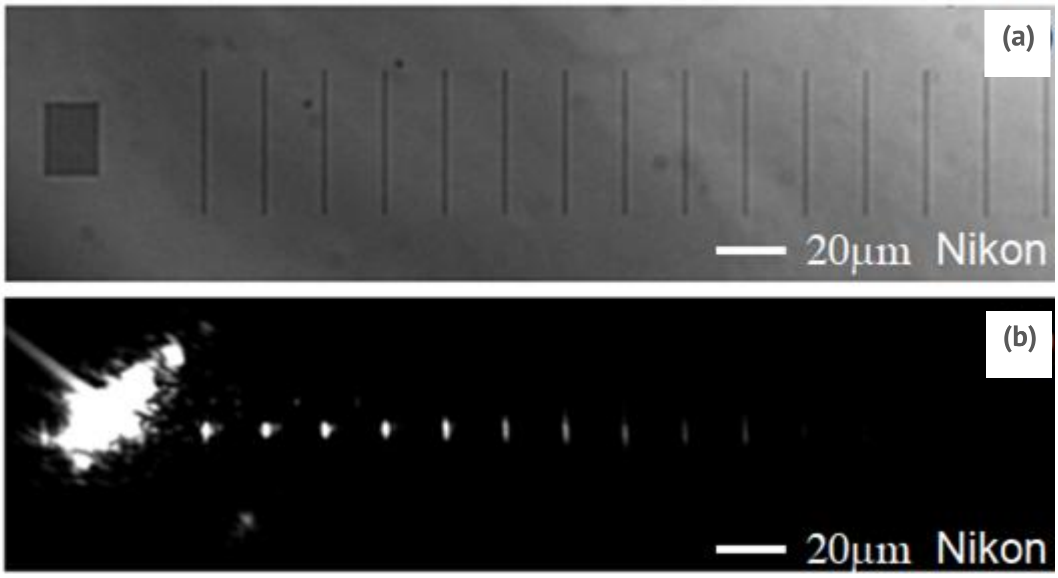

Supplementary Figure 13. | Optical microscopy of SPP[17]: (a) optical image of the Ag film surface with nanostructurescreated by a focused-ion-beam lithography, (b) optical image of the Ag film surface when SPP is excited by a laser radiation.

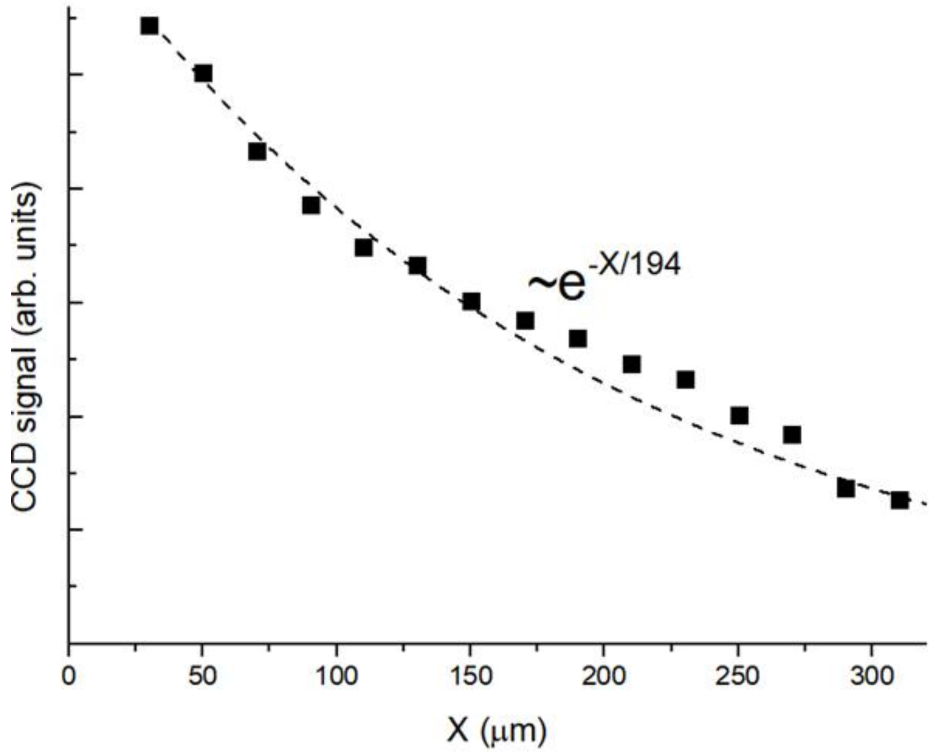

Supplementary Figure 14. | The decay curve of the SPP on an Ag thin film surface[17].

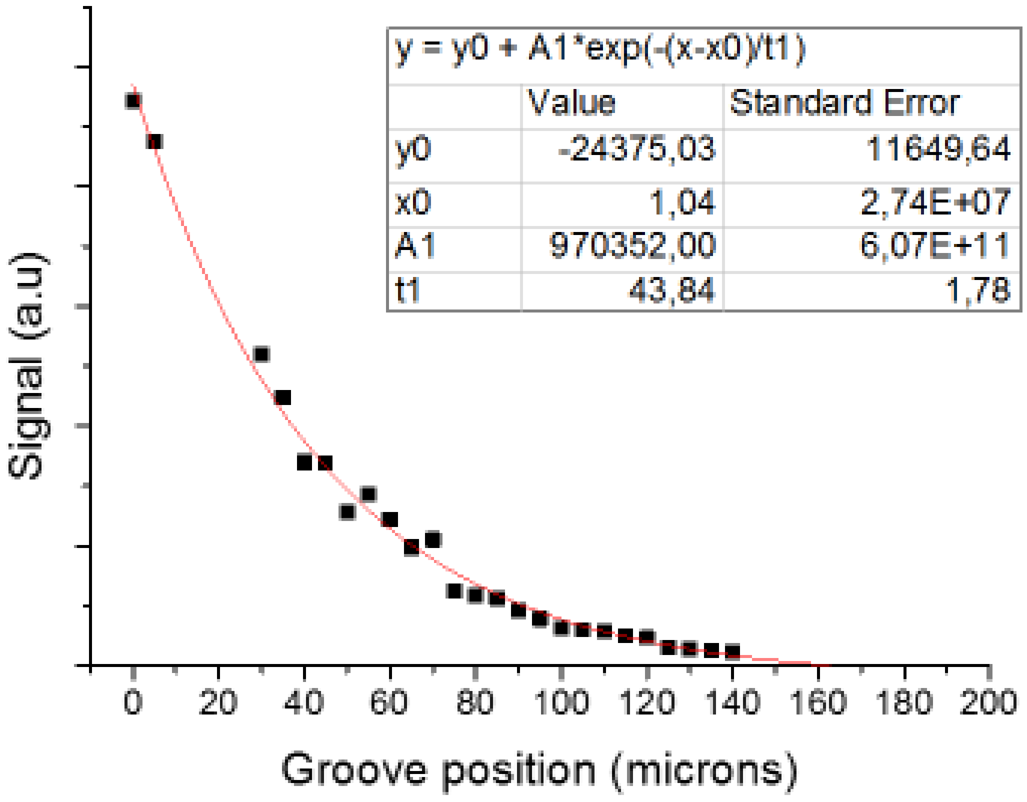

Supplementary Figure 15. | The decay curve of the SPP on an Au thin film surface[17].

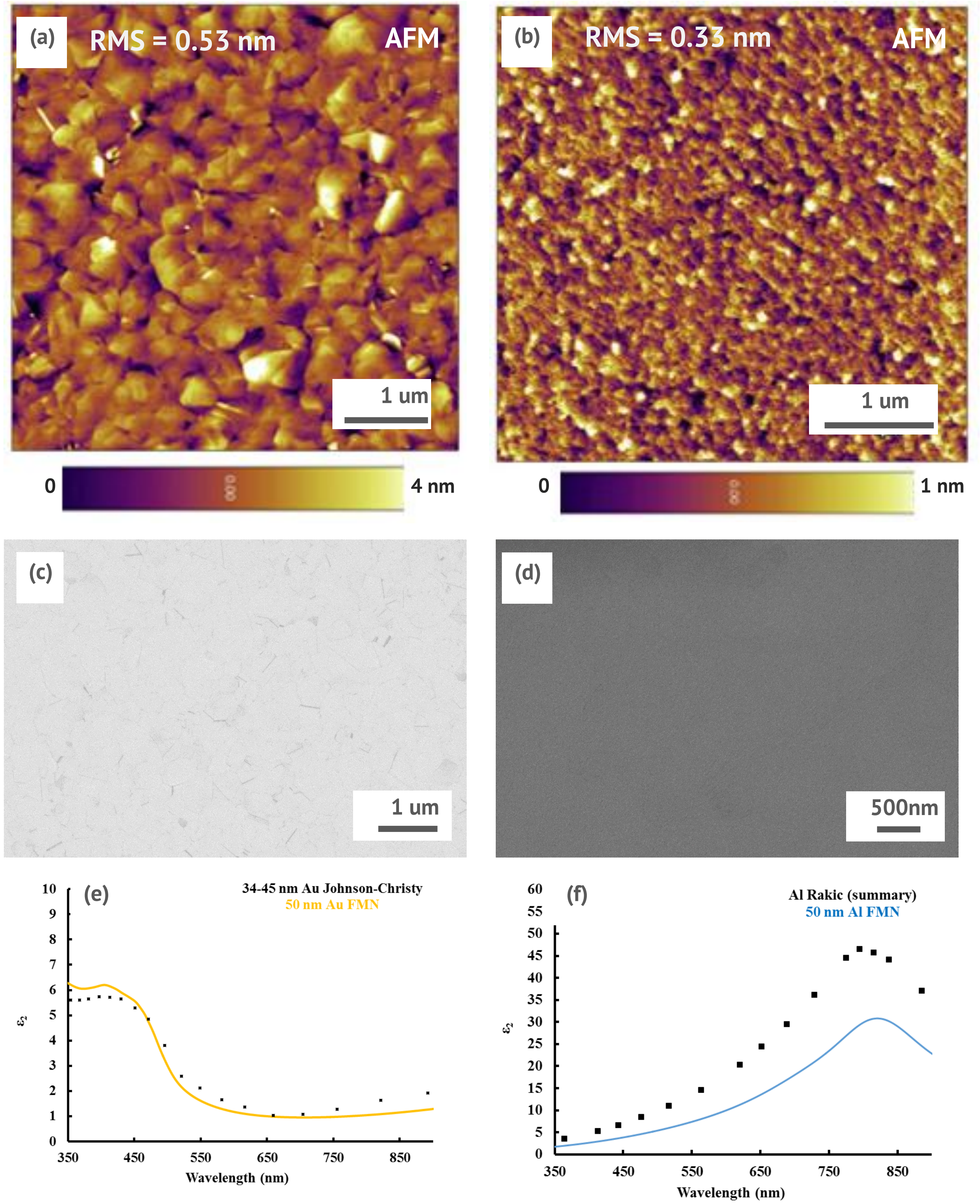

Supplementary Figure 16. | Al and Au thin films characterisation: (a) Au AFM scans, (b) Al AFM scans, (c) Au surface SEM scan, (d) Al surface SEM scan, (e) Au imaginary part of dielectric constant inverstigated by ellipsometry, (f) Al imaginary part of dielectric constant inverstigated by ellipsometry.

Supporting Table 1. | Thickness, surface roughness and microstructure of SCULL Ag films as a function of substrate type. AFM RMS roughness was determined from scans over a 2.5 × 2.5 μm^2 area. SP RMS roughness was determined from scans over a 20-μm length.

Substrate	Measured thickness [nm]	Crystalline structure	Average grain size, [nm]	Rocking curve Ag peak, FWHM [º]	AFM RMS roughness [nm]	Sample
Mica	35	Single-crystalline	no grains	not measured	0.35	M1
Si (111)	37	Single-crystalline	no grains	0.325	0.09	S1
Si (100)	39	Single-crystalline	no grains	0.829	0.28	S2
Si (110)	39	Single-crystalline	no grains	0.831	0.37	S3
Si (111)	68	Single-crystalline	no grains	0.221	0.36	S4
Si (111)	99	Single-crystalline	no grains	0.368	0.43	S5
Quartz	107	Nanocrystalline	less than 20	not measured	2.18	NC
Quartz	100	Polycrystalline	50	not measured	2.34	PC
Quartz	98	Polycrystalline	more than 500	not measured	2.22	PCBG

Supporting Table 2. | The silver thin films dielectric permittivity comparison

	Sample S1		Sample S2		Sample S3		Sample S4		Sample S5		Sample NC		Sample PC		Sample PCBG	
λ, nm	ε_1	ε_2	ε_1	ε_2	ε_1	ε_2	ε_1	ε_2	ε_1	ε_2	ε_1	ε_2	ε_1	ε_2	ε_1	ε_2
240.0	-0.440	3.8356	-0.259	3.5933	-0.425	3.7566	-0.175	4.0373	-0.474	3.4993	-0.095	2.9829	-0.207	3.1800	-0.333	3.4440
241.9	-0.363	3.8381	-0.251	3.6428	-0.354	3.7547	-0.172	3.9977	-0.456	3.6096	-0.053	2.9673	-0.227	3.1983	-0.347	3.4640
243.8	-0.300	3.8379	-0.236	3.6937	-0.302	3.7509	-0.177	3.9648	-0.409	3.7073	-0.028	2.9548	-0.243	3.2208	-0.357	3.4966
245.7	-0.246	3.8351	-0.214	3.7420	-0.263	3.7503	-0.187	3.9380	-0.336	3.7694	-0.013	2.9473	-0.261	3.2550	-0.358	3.5356
247.6	-0.200	3.8286	-0.187	3.7839	-0.232	3.7534	-0.201	3.9161	-0.268	3.7858	-0.004	2.9441	-0.261	3.3022	-0.350	3.5747
249.5	-0.164	3.8191	-0.157	3.8173	-0.206	3.7599	-0.221	3.8991	-0.230	3.7754	-0.001	2.9450	-0.244	3.3377	-0.336	3.6114
251.4	-0.141	3.8106	-0.130	3.8430	-0.184	3.7707	-0.246	3.8891	-0.223	3.7651	-0.001	2.9509	-0.233	3.3661	-0.317	3.6441
253.3	-0.128	3.8084	-0.109	3.8656	-0.164	3.7864	-0.276	3.8896	-0.234	3.7696	-0.003	2.9626	-0.211	3.3995	-0.296	3.6708
255.2	-0.121	3.8165	-0.092	3.8909	-0.143	3.8059	-0.307	3.9046	-0.249	3.7917	-0.004	2.9789	-0.189	3.4178	-0.278	3.6938
257.1	-0.114	3.8350	-0.074	3.9220	-0.120	3.8266	-0.333	3.9362	-0.259	3.8285	-0.005	2.9979	-0.168	3.4414	-0.263	3.7195
259.0	-0.105	3.8629	-0.048	3.9554	-0.100	3.8493	-0.346	3.9807	-0.256	3.8734	-0.009	3.0211	-0.137	3.4526	-0.245	3.7509
260.9	-0.091	3.9011	-0.016	3.9835	-0.081	3.8803	-0.343	4.0297	-0.241	3.9167	-0.014	3.0547	-0.124	3.4473	-0.217	3.7841
262.8	-0.067	3.9515	0.014	4.0055	-0.055	3.9257	-0.328	4.0776	-0.222	3.9526	-0.012	3.1044	-0.137	3.4594	-0.181	3.8079
264.7	-0.020	4.0095	0.038	4.0326	-0.008	3.9805	-0.306	4.1289	-0.209	3.9882	0.011	3.1658	-0.143	3.5055	-0.150	3.8197
266.6	0.054	4.0604	0.068	4.0751	0.066	4.0271	-0.269	4.1905	-0.199	4.0410	0.063	3.2221	-0.115	3.5673	-0.135	3.8382
268.5	0.147	4.0892	0.125	4.1234	0.155	4.0500	-0.200	4.2539	-0.168	4.1175	0.133	3.2562	-0.055	3.6126	-0.117	3.8827
270.4	0.243	4.0928	0.209	4.1510	0.243	4.0506	-0.098	4.2945	-0.094	4.1963	0.205	3.2661	0.009	3.6301	-0.062	3.9400
272.3	0.337	4.0775	0.292	4.1469	0.326	4.0403	0.015	4.2963	0.015	4.2433	0.269	3.2637	0.062	3.6410	0.026	3.9701
274.2	0.425	4.0446	0.357	4.1312	0.409	4.0192	0.117	4.2704	0.127	4.2512	0.333	3.2553	0.125	3.6606	0.108	3.9622
276.1	0.497	3.9951	0.420	4.1232	0.484	3.9799	0.213	4.2319	0.229	4.2419	0.399	3.2285	0.213	3.6560	0.167	3.9515
278.0	0.546	3.9459	0.494	4.1045	0.537	3.9378	0.309	4.1674	0.340	4.2214	0.444	3.1808	0.280	3.6139	0.237	3.9549
279.9	0.585	3.9145	0.554	4.0677	0.581	3.9147	0.373	4.0696	0.452	4.1573	0.458	3.1420	0.313	3.5846	0.324	3.9265
281.8	0.637	3.8948	0.598	4.0471	0.643	3.9025	0.388	3.9792	0.514	4.0644	0.464	3.1336	0.354	3.5823	0.371	3.8803
283.7	0.700	3.8681	0.662	4.0426	0.719	3.8781	0.382	3.9280	0.535	4.0002	0.491	3.1441	0.423	3.5759	0.406	3.8729
285.6	0.757	3.8375	0.749	4.0226	0.789	3.8432	0.391	3.9075	0.564	3.9724	0.539	3.1486	0.496	3.5426	0.479	3.8810
287.5	0.815	3.8265	0.831	3.9824	0.857	3.8226	0.421	3.8897	0.618	3.9476	0.586	3.1446	0.546	3.5024	0.575	3.8585
289.4	0.913	3.8316	0.906	3.9495	0.959	3.8147	0.448	3.8698	0.674	3.9057	0.637	3.1557	0.589	3.4921	0.654	3.8154
291.3	1.059	3.8061	1.008	3.9283	1.103	3.7768	0.472	3.8818	0.711	3.8746	0.724	3.1802	0.673	3.5055	0.724	3.7971
293.2	1.215	3.7299	1.147	3.8802	1.251	3.6924	0.549	3.9353	0.767	3.8867	0.856	3.1744	0.810	3.4890	0.841	3.7948
295.1	1.368	3.6320	1.290	3.7886	1.395	3.5914	0.712	3.9627	0.895	3.9042	0.994	3.1249	0.956	3.4210	1.005	3.7499
297.0	1.548	3.4917	1.429	3.6805	1.569	3.4534	0.905	3.9163	1.070	3.8631	1.139	3.0602	1.098	3.3305	1.168	3.6575
298.9	1.702	3.2718	1.597	3.5366	1.721	3.2330	1.100	3.8354	1.245	3.7765	1.316	2.9424	1.263	3.1991	1.344	3.5434
300.8	1.801	3.0304	1.744	3.3090	1.812	2.9903	1.339	3.6904	1.450	3.6560	1.454	2.7498	1.399	2.9933	1.539	3.3482
302.7	1.879	2.7726	1.826	3.0578	1.883	2.7376	1.533	3.4378	1.654	3.4319	1.549	2.5425	1.480	2.7727	1.664	3.0871
304.6	1.921	2.4758	1.883	2.8032	1.922	2.4492	1.664	3.1641	1.783	3.1601	1.625	2.3084	1.543	2.5436	1.743	2.8253
306.5	1.895	2.1609	1.914	2.5186	1.899	2.1393	1.772	2.8548	1.881	2.8744	1.652	2.0363	1.578	2.2803	1.801	2.5358
308.4	1.808	1.8665	1.888	2.2106	1.811	1.8470	1.815	2.4910	1.940	2.5411	1.609	1.7608	1.554	1.9976	1.800	2.2115
310.3	1.686	1.6039	1.798	1.9163	1.688	1.5883	1.763	2.1241	1.917	2.1812	1.516	1.5143	1.471	1.7323	1.723	1.8956
312.2	1.545	1.3633	1.669	1.6560	1.546	1.3546	1.646	1.7970	1.816	1.8468	1.398	1.2963	1.356	1.4974	1.598	1.6176
314.1	1.380	1.1388	1.523	1.4232	1.387	1.1369	1.499	1.5046	1.675	1.5525	1.262	1.0963	1.225	1.2840	1.453	1.3707
316.0	1.190	0.9367	1.361	1.2067	1.204	0.9378	1.323	1.2329	1.510	1.2841	1.101	0.9141	1.074	1.0846	1.289	1.1417
317.9	0.979	0.7663	1.179	1.0063	1.000	0.7664	1.112	0.9873	1.317	1.0357	0.918	0.7590	0.899	0.9035	1.098	0.9324
319.8	0.760	0.6309	0.975	0.8300	0.784	0.6279	0.870	0.7829	1.091	0.8179	0.721	0.6374	0.703	0.7503	0.884	0.7542

321.7	0.542	0.5272	0.757	0.6849	0.568	0.5210	0.616	0.6264	0.845	0.6423	0.523	0.5485	0.496	0.6302	0.657	0.6140
323.6	0.333	0.4488	0.536	0.5715	0.359	0.4402	0.365	0.5134	0.592	0.5104	0.331	0.4870	0.289	0.5417	0.431	0.5100
325.5	0.134	0.3895	0.320	0.4856	0.160	0.3796	0.126	0.4341	0.346	0.4162	0.148	0.4467	0.088	0.4798	0.214	0.4350
327.4	-0.054	0.3440	0.112	0.4212	-0.029	0.3337	-0.096	0.3791	0.113	0.3508	-0.023	0.4225	-0.103	0.4390	0.010	0.3816
329.3	-0.232	0.3086	-0.084	0.3730	-0.207	0.2984	-0.302	0.3408	-0.105	0.3069	-0.184	0.4104	-0.282	0.4145	-0.181	0.3435
331.2	-0.401	0.2806	-0.270	0.3365	-0.376	0.2710	-0.494	0.3141	-0.308	0.2784	-0.334	0.4075	-0.449	0.4027	-0.360	0.3161
333.1	-0.561	0.2581	-0.447	0.3087	-0.536	0.2493	-0.672	0.2956	-0.497	0.2607	-0.475	0.4114	-0.605	0.4005	-0.528	0.2962
335.0	-0.715	0.2399	-0.614	0.2873	-0.689	0.2318	-0.839	0.2830	-0.673	0.2504	-0.606	0.4205	-0.749	0.4055	-0.686	0.2816
336.9	-0.862	0.2250	-0.774	0.2708	-0.835	0.2176	-0.995	0.2743	-0.838	0.2454	-0.730	0.4334	-0.882	0.4156	-0.836	0.2709
338.8	-1.005	0.2128	-0.927	0.2580	-0.976	0.2059	-1.142	0.2676	-0.994	0.2447	-0.846	0.4495	-1.004	0.4288	-0.978	0.2628
340.8	-1.142	0.2029	-1.074	0.2480	-1.112	0.1961	-1.282	0.2619	-1.140	0.2474	-0.955	0.4681	-1.116	0.4433	-1.114	0.2567
342.7	-1.275	0.1949	-1.216	0.2404	-1.244	0.1879	-1.415	0.2565	-1.276	0.2520	-1.058	0.4889	-1.219	0.4574	-1.245	0.2521
344.6	-1.405	0.1884	-1.353	0.2345	-1.371	0.1808	-1.543	0.2512	-1.404	0.2561	-1.154	0.5111	-1.312	0.4693	-1.372	0.2485
346.5	-1.531	0.1832	-1.485	0.2301	-1.496	0.1745	-1.666	0.2455	-1.526	0.2587	-1.243	0.5335	-1.399	0.4775	-1.494	0.2457
348.4	-1.653	0.1790	-1.614	0.2268	-1.617	0.1688	-1.785	0.2386	-1.643	0.2600	-1.326	0.5549	-1.480	0.4811	-1.613	0.2435
350.3	-1.773	0.1757	-1.740	0.2245	-1.736	0.1637	-1.902	0.2307	-1.756	0.2604	-1.403	0.5745	-1.558	0.4795	-1.728	0.2416
352.2	-1.890	0.1730	-1.863	0.2230	-1.852	0.1590	-2.016	0.2222	-1.864	0.2585	-1.477	0.5919	-1.633	0.4725	-1.841	0.2401
354.1	-2.005	0.1709	-1.983	0.2222	-1.966	0.1545	-2.129	0.2130	-1.969	0.2530	-1.547	0.6073	-1.709	0.4606	-1.952	0.2388
356.0	-2.118	0.1691	-2.100	0.2218	-2.079	0.1502	-2.240	0.2030	-2.074	0.2453	-1.614	0.6207	-1.785	0.4443	-2.060	0.2377
357.9	-2.228	0.1673	-2.215	0.2219	-2.190	0.1461	-2.351	0.1923	-2.177	0.2365	-1.678	0.6314	-1.863	0.4245	-2.167	0.2367
359.8	-2.337	0.1655	-2.328	0.2221	-2.300	0.1420	-2.462	0.1814	-2.280	0.2260	-1.739	0.6389	-1.945	0.4023	-2.271	0.2357
361.7	-2.444	0.1636	-2.439	0.2223	-2.408	0.1379	-2.572	0.1707	-2.383	0.2132	-1.799	0.6428	-2.029	0.3788	-2.374	0.2347
363.6	-2.549	0.1614	-2.548	0.2225	-2.516	0.1339	-2.682	0.1606	-2.486	0.1989	-1.860	0.6437	-2.117	0.3552	-2.476	0.2336
365.5	-2.653	0.1589	-2.655	0.2225	-2.622	0.1300	-2.792	0.1510	-2.592	0.1844	-1.920	0.6423	-2.207	0.3321	-2.576	0.2324
367.4	-2.757	0.1560	-2.761	0.2222	-2.728	0.1261	-2.901	0.1419	-2.698	0.1707	-1.980	0.6388	-2.300	0.3102	-2.676	0.2311
369.3	-2.859	0.1528	-2.866	0.2217	-2.833	0.1222	-3.010	0.1333	-2.805	0.1578	-2.041	0.6332	-2.395	0.2900	-2.774	0.2296
371.2	-2.962	0.1492	-2.969	0.2210	-2.938	0.1184	-3.119	0.1253	-2.912	0.1456	-2.102	0.6253	-2.491	0.2718	-2.872	0.2280
373.1	-3.063	0.1453	-3.072	0.2198	-3.042	0.1146	-3.228	0.1180	-3.020	0.1339	-2.165	0.6154	-2.589	0.2559	-2.969	0.2264
375.0	-3.165	0.1411	-3.174	0.2183	-3.146	0.1109	-3.336	0.1115	-3.128	0.1228	-2.230	0.6041	-2.687	0.2422	-3.065	0.2246
376.9	-3.266	0.1366	-3.275	0.2164	-3.249	0.1073	-3.444	0.1059	-3.236	0.1127	-2.297	0.5922	-2.785	0.2306	-3.161	0.2227
378.8	-3.367	0.1320	-3.375	0.2140	-3.352	0.1039	-3.551	0.1010	-3.345	0.1039	-2.366	0.5802	-2.882	0.2210	-3.257	0.2208
380.7	-3.468	0.1274	-3.476	0.2112	-3.455	0.1007	-3.658	0.0969	-3.454	0.0964	-2.437	0.5687	-2.980	0.2130	-3.352	0.2187
382.6	-3.569	0.1227	-3.576	0.2080	-3.558	0.0976	-3.765	0.0934	-3.563	0.0903	-2.509	0.5577	-3.076	0.2063	-3.446	0.2166
384.5	-3.670	0.1182	-3.675	0.2045	-3.660	0.0947	-3.870	0.0904	-3.671	0.0853	-2.582	0.5474	-3.172	0.2008	-3.540	0.2143
386.4	-3.772	0.1138	-3.775	0.2007	-3.763	0.0919	-3.975	0.0877	-3.778	0.0814	-2.655	0.5376	-3.267	0.1962	-3.634	0.2120
388.3	-3.873	0.1096	-3.875	0.1967	-3.865	0.0892	-4.080	0.0855	-3.885	0.0783	-2.730	0.5284	-3.362	0.1923	-3.728	0.2095
390.2	-3.975	0.1056	-3.974	0.1923	-3.967	0.0867	-4.184	0.0835	-3.991	0.0758	-2.805	0.5198	-3.456	0.1891	-3.822	0.2070
392.1	-4.076	0.1018	-4.074	0.1877	-4.069	0.0844	-4.288	0.0817	-4.097	0.0737	-2.881	0.5119	-3.549	0.1864	-3.916	0.2044
394.0	-4.178	0.0982	-4.174	0.1830	-4.171	0.0822	-4.391	0.0801	-4.202	0.0721	-2.958	0.5049	-3.642	0.1841	-4.009	0.2017
395.9	-4.280	0.0950	-4.274	0.1780	-4.272	0.0802	-4.494	0.0787	-4.306	0.0708	-3.036	0.4990	-3.734	0.1822	-4.103	0.1989
397.8	-4.382	0.0920	-4.374	0.1731	-4.374	0.0784	-4.596	0.0774	-4.410	0.0697	-3.113	0.4941	-3.826	0.1805	-4.196	0.1962
399.7	-4.484	0.0893	-4.475	0.1681	-4.476	0.0767	-4.698	0.0763	-4.513	0.0688	-3.191	0.4904	-3.917	0.1791	-4.290	0.1934
401.6	-4.585	0.0869	-4.576	0.1632	-4.577	0.0752	-4.800	0.0752	-4.616	0.0680	-3.269	0.4876	-4.009	0.1779	-4.384	0.1906
403.5	-4.687	0.0847	-4.677	0.1584	-4.679	0.0738	-4.902	0.0742	-4.719	0.0674	-3.347	0.4858	-4.099	0.1769	-4.477	0.1879
405.4	-4.789	0.0828	-4.778	0.1537	-4.781	0.0725	-5.004	0.0733	-4.821	0.0669	-3.424	0.4848	-4.190	0.1761	-4.571	0.1852
407.3	-4.891	0.0811	-4.880	0.1492	-4.882	0.0714	-5.105	0.0725	-4.923	0.0664	-3.501	0.4845	-4.280	0.1754	-4.665	0.1826

409.2	-4.993	0.0796	-4.982	0.1447	-4.984	0.0703	-5.207	0.0718	-5.025	0.0661	-3.577	0.4847	-4.371	0.1748	-4.759	0.1800
411.1	-5.094	0.0783	-5.084	0.1404	-5.085	0.0694	-5.308	0.0711	-5.127	0.0658	-3.653	0.4853	-4.461	0.1744	-4.854	0.1775
413.0	-5.196	0.0771	-5.187	0.1362	-5.187	0.0686	-5.409	0.0704	-5.229	0.0656	-3.728	0.4862	-4.551	0.1740	-4.948	0.1751
414.9	-5.298	0.0761	-5.289	0.1322	-5.288	0.0678	-5.510	0.0698	-5.331	0.0654	-3.803	0.4874	-4.641	0.1738	-5.042	0.1727
416.8	-5.400	0.0751	-5.392	0.1283	-5.390	0.0671	-5.611	0.0693	-5.432	0.0653	-3.877	0.4886	-4.730	0.1736	-5.137	0.1704
418.7	-5.502	0.0743	-5.495	0.1247	-5.492	0.0664	-5.713	0.0688	-5.534	0.0652	-3.951	0.4899	-4.820	0.1735	-5.232	0.1681
420.6	-5.603	0.0735	-5.599	0.1212	-5.594	0.0659	-5.814	0.0683	-5.635	0.0651	-4.025	0.4911	-4.910	0.1734	-5.327	0.1659
422.5	-5.705	0.0728	-5.702	0.1179	-5.696	0.0653	-5.915	0.0679	-5.737	0.0651	-4.098	0.4922	-5.000	0.1734	-5.422	0.1638
424.4	-5.807	0.0722	-5.806	0.1148	-5.798	0.0649	-6.016	0.0675	-5.838	0.0651	-4.171	0.4931	-5.089	0.1735	-5.517	0.1617
426.3	-5.910	0.0717	-5.910	0.1120	-5.900	0.0644	-6.118	0.0671	-5.939	0.0651	-4.244	0.4939	-5.179	0.1736	-5.612	0.1596
428.2	-6.012	0.0712	-6.015	0.1094	-6.002	0.0640	-6.219	0.0668	-6.041	0.0652	-4.316	0.4944	-5.269	0.1738	-5.708	0.1577
430.1	-6.114	0.0707	-6.119	0.1070	-6.104	0.0637	-6.321	0.0664	-6.143	0.0653	-4.389	0.4947	-5.359	0.1741	-5.804	0.1558
432.0	-6.217	0.0703	-6.224	0.1048	-6.207	0.0634	-6.422	0.0662	-6.244	0.0654	-4.461	0.4947	-5.448	0.1743	-5.900	0.1539
433.9	-6.319	0.0700	-6.328	0.1028	-6.310	0.0631	-6.524	0.0659	-6.346	0.0655	-4.533	0.4944	-5.538	0.1747	-5.996	0.1522
435.8	-6.422	0.0697	-6.433	0.1009	-6.413	0.0628	-6.626	0.0657	-6.448	0.0657	-4.606	0.4939	-5.628	0.1750	-6.092	0.1505
437.7	-6.525	0.0694	-6.538	0.0992	-6.516	0.0626	-6.728	0.0655	-6.550	0.0658	-4.678	0.4930	-5.719	0.1754	-6.189	0.1489
439.6	-6.628	0.0692	-6.644	0.0977	-6.619	0.0624	-6.830	0.0653	-6.652	0.0660	-4.751	0.4919	-5.809	0.1759	-6.286	0.1473
441.5	-6.731	0.0689	-6.749	0.0963	-6.722	0.0622	-6.932	0.0651	-6.754	0.0662	-4.824	0.4905	-5.899	0.1763	-6.383	0.1459
443.4	-6.835	0.0688	-6.855	0.0950	-6.826	0.0621	-7.035	0.0650	-6.856	0.0665	-4.896	0.4889	-5.990	0.1768	-6.480	0.1445
445.3	-6.939	0.0686	-6.960	0.0939	-6.930	0.0620	-7.137	0.0649	-6.959	0.0667	-4.969	0.4870	-6.080	0.1774	-6.578	0.1433
447.2	-7.042	0.0685	-7.066	0.0928	-7.034	0.0619	-7.240	0.0648	-7.061	0.0670	-5.043	0.4848	-6.171	0.1779	-6.676	0.1421
449.1	-7.147	0.0684	-7.172	0.0918	-7.138	0.0618	-7.343	0.0647	-7.164	0.0672	-5.116	0.4825	-6.262	0.1785	-6.774	0.1410
451.0	-7.251	0.0683	-7.279	0.0910	-7.243	0.0617	-7.446	0.0647	-7.267	0.0675	-5.190	0.4798	-6.353	0.1792	-6.872	0.1400
452.9	-7.355	0.0683	-7.385	0.0902	-7.347	0.0617	-7.549	0.0646	-7.370	0.0678	-5.264	0.4770	-6.444	0.1798	-6.971	0.1391
454.8	-7.460	0.0683	-7.492	0.0894	-7.452	0.0617	-7.653	0.0646	-7.473	0.0681	-5.338	0.4739	-6.536	0.1805	-7.069	0.1383
456.7	-7.565	0.0683	-7.598	0.0888	-7.558	0.0617	-7.757	0.0646	-7.577	0.0684	-5.413	0.4706	-6.627	0.1812	-7.168	0.1375
458.6	-7.671	0.0683	-7.705	0.0882	-7.663	0.0617	-7.861	0.0646	-7.681	0.0688	-5.488	0.4671	-6.719	0.1819	-7.267	0.1369
460.5	-7.776	0.0683	-7.813	0.0876	-7.769	0.0617	-7.965	0.0646	-7.785	0.0691	-5.563	0.4634	-6.811	0.1827	-7.367	0.1363
462.4	-7.882	0.0684	-7.920	0.0871	-7.875	0.0618	-8.070	0.0647	-7.889	0.0695	-5.639	0.4595	-6.903	0.1835	-7.466	0.1358
464.3	-7.988	0.0685	-8.028	0.0866	-7.981	0.0619	-8.174	0.0647	-7.993	0.0699	-5.715	0.4555	-6.996	0.1843	-7.566	0.1353
466.2	-8.094	0.0686	-8.135	0.0862	-8.088	0.0619	-8.279	0.0648	-8.098	0.0702	-5.791	0.4513	-7.088	0.1851	-7.666	0.1350
468.1	-8.201	0.0687	-8.244	0.0858	-8.195	0.0620	-8.385	0.0649	-8.203	0.0706	-5.868	0.4470	-7.181	0.1860	-7.767	0.1347
470.0	-8.308	0.0688	-8.352	0.0855	-8.302	0.0622	-8.490	0.0650	-8.308	0.0710	-5.946	0.4426	-7.274	0.1869	-7.867	0.1344
471.9	-8.415	0.0690	-8.461	0.0852	-8.409	0.0623	-8.596	0.0651	-8.413	0.0715	-6.023	0.4381	-7.367	0.1878	-7.968	0.1342
473.8	-8.522	0.0691	-8.569	0.0849	-8.517	0.0624	-8.702	0.0652	-8.519	0.0719	-6.102	0.4335	-7.461	0.1887	-8.069	0.1341
475.7	-8.630	0.0693	-8.679	0.0846	-8.625	0.0626	-8.808	0.0654	-8.624	0.0723	-6.180	0.4288	-7.555	0.1896	-8.171	0.1340
477.6	-8.738	0.0695	-8.788	0.0844	-8.733	0.0628	-8.915	0.0655	-8.730	0.0728	-6.259	0.4241	-7.649	0.1906	-8.272	0.1340
479.5	-8.847	0.0698	-8.898	0.0842	-8.842	0.0630	-9.021	0.0657	-8.837	0.0732	-6.339	0.4194	-7.743	0.1916	-8.374	0.1340
481.4	-8.955	0.0700	-9.007	0.0840	-8.951	0.0632	-9.129	0.0658	-8.943	0.0737	-6.419	0.4146	-7.837	0.1926	-8.476	0.1341
483.3	-9.064	0.0702	-9.118	0.0839	-9.060	0.0634	-9.236	0.0660	-9.050	0.0742	-6.499	0.4099	-7.932	0.1936	-8.579	0.1342
485.2	-9.173	0.0705	-9.228	0.0837	-9.170	0.0636	-9.344	0.0662	-9.157	0.0746	-6.580	0.4051	-8.027	0.1947	-8.681	0.1344
487.1	-9.283	0.0708	-9.339	0.0836	-9.279	0.0638	-9.452	0.0665	-9.265	0.0751	-6.661	0.4004	-8.122	0.1957	-8.784	0.1346
489.0	-9.393	0.0711	-9.450	0.0835	-9.390	0.0641	-9.560	0.0667	-9.372	0.0756	-6.743	0.3957	-8.217	0.1968	-8.887	0.1349
490.9	-9.503	0.0714	-9.561	0.0835	-9.500	0.0643	-9.668	0.0669	-9.480	0.0762	-6.825	0.3910	-8.313	0.1979	-8.991	0.1351
492.8	-9.614	0.0717	-9.673	0.0834	-9.611	0.0646	-9.777	0.0672	-9.589	0.0767	-6.908	0.3864	-8.409	0.1991	-9.095	0.1355
494.7	-9.724	0.0720	-9.785	0.0834	-9.722	0.0649	-9.886	0.0674	-9.697	0.0772	-6.991	0.3818	-8.505	0.2002	-9.198	0.1358

496.6	-9.836	0.0724	-9.897	0.0833	-9.833	0.0652	-9.996	0.0677	-9.806	0.0777	-7.074	0.3773	-8.601	0.2014	-9.303	0.1362
498.5	-9.947	0.0728	-10.010	0.0833	-9.945	0.0655	-10.106	0.0680	-9.915	0.0783	-7.158	0.3728	-8.698	0.2026	-9.407	0.1366
500.4	-10.059	0.0731	-10.122	0.0834	-10.057	0.0658	-10.216	0.0683	-10.024	0.0788	-7.242	0.3684	-8.795	0.2038	-9.512	0.1370
502.3	-10.171	0.0735	-10.236	0.0834	-10.170	0.0662	-10.326	0.0686	-10.134	0.0794	-7.327	0.3640	-8.892	0.2050	-9.617	0.1375
504.2	-10.284	0.0739	-10.349	0.0834	-10.283	0.0665	-10.437	0.0689	-10.244	0.0800	-7.412	0.3597	-8.990	0.2062	-9.723	0.1380
506.1	-10.396	0.0744	-10.463	0.0835	-10.396	0.0669	-10.548	0.0692	-10.354	0.0806	-7.497	0.3554	-9.088	0.2075	-9.828	0.1385
508.0	-10.510	0.0748	-10.577	0.0836	-10.509	0.0672	-10.659	0.0695	-10.465	0.0812	-7.583	0.3513	-9.186	0.2088	-9.934	0.1391
509.9	-10.623	0.0752	-10.691	0.0837	-10.623	0.0676	-10.771	0.0699	-10.576	0.0818	-7.669	0.3471	-9.284	0.2100	-10.041	0.1396
511.8	-10.737	0.0757	-10.806	0.0838	-10.737	0.0680	-10.883	0.0702	-10.687	0.0824	-7.756	0.3431	-9.383	0.2114	-10.147	0.1402
513.7	-10.851	0.0762	-10.921	0.0839	-10.852	0.0684	-10.995	0.0706	-10.799	0.0830	-7.842	0.3391	-9.482	0.2127	-10.254	0.1408
515.6	-10.966	0.0766	-11.037	0.0840	-10.967	0.0688	-11.108	0.0710	-10.911	0.0836	-7.930	0.3353	-9.581	0.2140	-10.361	0.1415
517.5	-11.081	0.0771	-11.152	0.0841	-11.082	0.0692	-11.221	0.0714	-11.023	0.0842	-8.017	0.3315	-9.680	0.2154	-10.469	0.1421
519.4	-11.196	0.0776	-11.269	0.0843	-11.198	0.0696	-11.334	0.0718	-11.135	0.0849	-8.106	0.3278	-9.780	0.2168	-10.577	0.1428
521.3	-11.312	0.0782	-11.385	0.0844	-11.314	0.0701	-11.448	0.0722	-11.248	0.0855	-8.194	0.3241	-9.880	0.2182	-10.685	0.1435
523.2	-11.428	0.0787	-11.502	0.0846	-11.430	0.0705	-11.562	0.0726	-11.361	0.0862	-8.283	0.3206	-9.981	0.2196	-10.793	0.1442
525.1	-11.544	0.0792	-11.619	0.0848	-11.547	0.0710	-11.676	0.0730	-11.475	0.0868	-8.372	0.3172	-10.082	0.2210	-10.902	0.1449
527.0	-11.661	0.0798	-11.736	0.0850	-11.664	0.0714	-11.791	0.0735	-11.589	0.0875	-8.462	0.3138	-10.183	0.2225	-11.011	0.1457
528.9	-11.778	0.0804	-11.854	0.0852	-11.781	0.0719	-11.906	0.0739	-11.703	0.0882	-8.552	0.3106	-10.284	0.2240	-11.120	0.1464
530.8	-11.896	0.0810	-11.972	0.0854	-11.899	0.0724	-12.022	0.0744	-11.817	0.0889	-8.642	0.3074	-10.385	0.2254	-11.230	0.1472
532.7	-12.013	0.0816	-12.091	0.0857	-12.017	0.0729	-12.137	0.0748	-11.932	0.0896	-8.732	0.3044	-10.487	0.2270	-11.340	0.1480
534.6	-12.132	0.0822	-12.210	0.0859	-12.135	0.0734	-12.253	0.0753	-12.047	0.0903	-8.823	0.3015	-10.590	0.2285	-11.450	0.1488
536.5	-12.250	0.0828	-12.329	0.0861	-12.254	0.0739	-12.370	0.0758	-12.163	0.0910	-8.915	0.2986	-10.692	0.2300	-11.561	0.1497
538.4	-12.369	0.0834	-12.448	0.0864	-12.374	0.0745	-12.487	0.0763	-12.279	0.0917	-9.007	0.2959	-10.795	0.2316	-11.672	0.1505
540.4	-12.489	0.0841	-12.568	0.0867	-12.493	0.0750	-12.604	0.0768	-12.395	0.0925	-9.099	0.2933	-10.898	0.2331	-11.783	0.1514
542.3	-12.608	0.0847	-12.689	0.0870	-12.613	0.0756	-12.721	0.0773	-12.511	0.0932	-9.191	0.2908	-11.002	0.2347	-11.895	0.1523
544.2	-12.728	0.0854	-12.809	0.0872	-12.734	0.0761	-12.839	0.0778	-12.628	0.0939	-9.284	0.2884	-11.105	0.2364	-12.007	0.1532
546.1	-12.849	0.0861	-12.930	0.0876	-12.854	0.0767	-12.958	0.0784	-12.745	0.0947	-9.377	0.2861	-11.210	0.2380	-12.119	0.1541
548.0	-12.970	0.0868	-13.052	0.0879	-12.976	0.0773	-13.076	0.0789	-12.863	0.0955	-9.470	0.2839	-11.314	0.2396	-12.232	0.1550
549.9	-13.091	0.0875	-13.173	0.0882	-13.097	0.0779	-13.195	0.0795	-12.981	0.0962	-9.564	0.2818	-11.419	0.2413	-12.345	0.1559
551.8	-13.213	0.0882	-13.296	0.0885	-13.219	0.0785	-13.315	0.0800	-13.099	0.0970	-9.658	0.2799	-11.524	0.2430	-12.458	0.1569
553.7	-13.335	0.0889	-13.418	0.0889	-13.341	0.0791	-13.434	0.0806	-13.218	0.0978	-9.752	0.2780	-11.629	0.2447	-12.572	0.1578
555.6	-13.457	0.0897	-13.541	0.0892	-13.464	0.0797	-13.554	0.0812	-13.337	0.0986	-9.847	0.2762	-11.735	0.2464	-12.686	0.1588
557.5	-13.580	0.0904	-13.664	0.0896	-13.587	0.0804	-13.675	0.0818	-13.456	0.0994	-9.942	0.2746	-11.841	0.2481	-12.800	0.1598
559.4	-13.703	0.0912	-13.788	0.0899	-13.711	0.0810	-13.796	0.0824	-13.576	0.1002	-10.037	0.2730	-11.947	0.2499	-12.915	0.1608
561.3	-13.826	0.0920	-13.912	0.0903	-13.834	0.0816	-13.917	0.0830	-13.696	0.1010	-10.133	0.2716	-12.054	0.2516	-13.030	0.1619
563.2	-13.950	0.0928	-14.036	0.0907	-13.959	0.0823	-14.038	0.0836	-13.816	0.1019	-10.229	0.2702	-12.161	0.2534	-13.145	0.1629
565.1	-14.075	0.0936	-14.161	0.0911	-14.083	0.0830	-14.160	0.0842	-13.937	0.1027	-10.325	0.2689	-12.268	0.2552	-13.261	0.1639
567.0	-14.200	0.0944	-14.286	0.0915	-14.208	0.0837	-14.283	0.0849	-14.058	0.1035	-10.421	0.2677	-12.376	0.2571	-13.377	0.1650
568.9	-14.325	0.0952	-14.411	0.0919	-14.334	0.0844	-14.405	0.0855	-14.179	0.1044	-10.518	0.2666	-12.484	0.2589	-13.494	0.1661
570.8	-14.450	0.0961	-14.537	0.0924	-14.460	0.0851	-14.528	0.0862	-14.301	0.1052	-10.615	0.2656	-12.592	0.2608	-13.610	0.1672
572.7	-14.576	0.0970	-14.663	0.0928	-14.586	0.0858	-14.652	0.0869	-14.423	0.1061	-10.713	0.2647	-12.701	0.2626	-13.728	0.1683
574.6	-14.703	0.0978	-14.790	0.0932	-14.712	0.0865	-14.776	0.0876	-14.546	0.1070	-10.810	0.2639	-12.810	0.2645	-13.845	0.1694
576.5	-14.829	0.0987	-14.917	0.0937	-14.839	0.0873	-14.900	0.0883	-14.669	0.1079	-10.908	0.2631	-12.919	0.2664	-13.963	0.1705
578.4	-14.956	0.0996	-15.044	0.0942	-14.967	0.0880	-15.025	0.0890	-14.792	0.1088	-11.007	0.2624	-13.029	0.2684	-14.081	0.1716
580.3	-15.084	0.1005	-15.172	0.0946	-15.095	0.0888	-15.150	0.0897	-14.915	0.1097	-11.105	0.2618	-13.139	0.2703	-14.200	0.1728
582.2	-15.212	0.1015	-15.300	0.0951	-15.223	0.0895	-15.275	0.0904	-15.039	0.1106	-11.204	0.2613	-13.249	0.2723	-14.319	0.1740

584.1	-15.340	0.1024	-15.429	0.0956	-15.351	0.0903	-15.401	0.0911	-15.164	0.1115	-11.303	0.2609	-13.360	0.2743	-14.438	0.1751
586.0	-15.469	0.1033	-15.558	0.0961	-15.480	0.0911	-15.527	0.0919	-15.289	0.1124	-11.403	0.2605	-13.471	0.2763	-14.558	0.1763
587.9	-15.598	0.1043	-15.687	0.0966	-15.610	0.0919	-15.653	0.0926	-15.414	0.1134	-11.502	0.2601	-13.582	0.2783	-14.678	0.1775
589.8	-15.728	0.1053	-15.817	0.0971	-15.740	0.0927	-15.780	0.0934	-15.539	0.1143	-11.602	0.2599	-13.694	0.2803	-14.798	0.1787
591.7	-15.858	0.1063	-15.947	0.0977	-15.870	0.0935	-15.908	0.0942	-15.665	0.1153	-11.703	0.2597	-13.806	0.2824	-14.919	0.1800
593.6	-15.988	0.1073	-16.077	0.0982	-16.001	0.0944	-16.035	0.0950	-15.791	0.1162	-11.803	0.2596	-13.918	0.2845	-15.040	0.1812
595.5	-16.119	0.1083	-16.208	0.0987	-16.132	0.0952	-16.163	0.0957	-15.918	0.1172	-11.904	0.2595	-14.031	0.2866	-15.161	0.1825
597.4	-16.250	0.1094	-16.339	0.0993	-16.263	0.0961	-16.292	0.0966	-16.045	0.1182	-12.006	0.2595	-14.144	0.2887	-15.283	0.1837
599.3	-16.382	0.1104	-16.471	0.0999	-16.395	0.0969	-16.421	0.0974	-16.172	0.1191	-12.107	0.2596	-14.257	0.2908	-15.405	0.1850
601.2	-16.514	0.1115	-16.603	0.1004	-16.527	0.0978	-16.550	0.0982	-16.300	0.1201	-12.209	0.2597	-14.371	0.2930	-15.528	0.1863
603.1	-16.646	0.1126	-16.735	0.1010	-16.660	0.0987	-16.680	0.0990	-16.428	0.1211	-12.311	0.2598	-14.485	0.2951	-15.651	0.1876
605.0	-16.779	0.1136	-16.868	0.1016	-16.793	0.0996	-16.810	0.0999	-16.556	0.1222	-12.413	0.2600	-14.599	0.2973	-15.774	0.1889
606.9	-16.913	0.1148	-17.002	0.1022	-16.926	0.1005	-16.940	0.1007	-16.685	0.1232	-12.516	0.2603	-14.714	0.2995	-15.898	0.1903
608.8	-17.046	0.1159	-17.135	0.1028	-17.060	0.1015	-17.071	0.1016	-16.814	0.1242	-12.619	0.2606	-14.829	0.3018	-16.022	0.1916
610.7	-17.181	0.1170	-17.269	0.1034	-17.195	0.1024	-17.203	0.1025	-16.944	0.1253	-12.722	0.2610	-14.945	0.3040	-16.146	0.1930
612.6	-17.315	0.1182	-17.404	0.1041	-17.330	0.1033	-17.334	0.1034	-17.074	0.1263	-12.826	0.2614	-15.061	0.3063	-16.271	0.1943
614.5	-17.450	0.1193	-17.539	0.1047	-17.465	0.1043	-17.466	0.1043	-17.204	0.1274	-12.930	0.2618	-15.177	0.3086	-16.396	0.1957
616.4	-17.586	0.1205	-17.674	0.1054	-17.600	0.1053	-17.599	0.1052	-17.335	0.1284	-13.034	0.2623	-15.293	0.3109	-16.522	0.1971
618.3	-17.721	0.1217	-17.809	0.1060	-17.736	0.1063	-17.732	0.1061	-17.466	0.1295	-13.138	0.2629	-15.410	0.3132	-16.648	0.1985
620.2	-17.858	0.1229	-17.946	0.1067	-17.873	0.1073	-17.865	0.1071	-17.598	0.1306	-13.243	0.2634	-15.527	0.3155	-16.774	0.1999
622.1	-17.994	0.1242	-18.082	0.1074	-18.010	0.1083	-17.999	0.1080	-17.730	0.1317	-13.348	0.2641	-15.645	0.3179	-16.901	0.2014
624.0	-18.131	0.1254	-18.219	0.1080	-18.147	0.1093	-18.133	0.1090	-17.862	0.1328	-13.453	0.2647	-15.763	0.3203	-17.028	0.2028
625.9	-18.269	0.1267	-18.356	0.1087	-18.285	0.1103	-18.267	0.1100	-17.995	0.1339	-13.559	0.2654	-15.881	0.3227	-17.155	0.2043
627.8	-18.407	0.1279	-18.494	0.1094	-18.423	0.1114	-18.402	0.1110	-18.128	0.1350	-13.665	0.2662	-16.000	0.3251	-17.283	0.2058
629.7	-18.545	0.1292	-18.632	0.1102	-18.561	0.1124	-18.538	0.1119	-18.261	0.1361	-13.771	0.2669	-16.119	0.3276	-17.411	0.2073
631.6	-18.684	0.1305	-18.770	0.1109	-18.700	0.1135	-18.673	0.1130	-18.395	0.1373	-13.878	0.2677	-16.238	0.3300	-17.539	0.2088
633.5	-18.823	0.1319	-18.909	0.1116	-18.840	0.1146	-18.809	0.1140	-18.529	0.1384	-13.985	0.2686	-16.358	0.3325	-17.668	0.2103
635.4	-18.963	0.1332	-19.049	0.1124	-18.979	0.1157	-18.946	0.1150	-18.664	0.1396	-14.092	0.2695	-16.478	0.3350	-17.798	0.2118
637.3	-19.103	0.1346	-19.188	0.1131	-19.120	0.1168	-19.083	0.1161	-18.799	0.1408	-14.199	0.2704	-16.599	0.3376	-17.927	0.2133
639.2	-19.244	0.1359	-19.329	0.1139	-19.260	0.1179	-19.220	0.1171	-18.934	0.1419	-14.307	0.2713	-16.719	0.3401	-18.058	0.2149
641.1	-19.385	0.1373	-19.469	0.1147	-19.401	0.1191	-19.358	0.1182	-19.070	0.1431	-14.415	0.2723	-16.841	0.3427	-18.188	0.2165
643.0	-19.526	0.1387	-19.610	0.1154	-19.543	0.1202	-19.496	0.1193	-19.206	0.1443	-14.524	0.2733	-16.962	0.3453	-18.319	0.2181
644.9	-19.668	0.1402	-19.751	0.1162	-19.685	0.1214	-19.635	0.1204	-19.343	0.1455	-14.632	0.2744	-17.084	0.3479	-18.450	0.2196
646.8	-19.810	0.1416	-19.893	0.1170	-19.827	0.1225	-19.774	0.1215	-19.480	0.1467	-14.741	0.2754	-17.206	0.3505	-18.582	0.2213
648.7	-19.953	0.1431	-20.036	0.1179	-19.970	0.1237	-19.913	0.1226	-19.617	0.1480	-14.851	0.2766	-17.329	0.3532	-18.714	0.2229
650.6	-20.096	0.1445	-20.178	0.1187	-20.113	0.1249	-20.053	0.1237	-19.755	0.1492	-14.960	0.2777	-17.452	0.3559	-18.846	0.2245
652.5	-20.240	0.1460	-20.321	0.1195	-20.257	0.1262	-20.194	0.1249	-19.893	0.1504	-15.070	0.2789	-17.575	0.3585	-18.979	0.2262
654.4	-20.384	0.1476	-20.465	0.1204	-20.401	0.1274	-20.334	0.1260	-20.032	0.1517	-15.181	0.2801	-17.699	0.3613	-19.112	0.2278
656.3	-20.528	0.1491	-20.609	0.1212	-20.546	0.1286	-20.475	0.1272	-20.171	0.1530	-15.291	0.2813	-17.823	0.3640	-19.246	0.2295
658.2	-20.673	0.1506	-20.753	0.1221	-20.691	0.1299	-20.617	0.1284	-20.310	0.1542	-15.402	0.2825	-17.948	0.3668	-19.380	0.2312
660.1	-20.819	0.1522	-20.898	0.1229	-20.836	0.1312	-20.759	0.1296	-20.450	0.1555	-15.513	0.2838	-18.072	0.3696	-19.514	0.2329
662.0	-20.964	0.1538	-21.043	0.1238	-20.982	0.1325	-20.901	0.1308	-20.590	0.1568	-15.625	0.2851	-18.198	0.3724	-19.649	0.2347
663.9	-21.111	0.1554	-21.188	0.1247	-21.128	0.1338	-21.044	0.1320	-20.730	0.1581	-15.737	0.2865	-18.323	0.3752	-19.784	0.2364
665.8	-21.257	0.1570	-21.334	0.1256	-21.275	0.1351	-21.187	0.1332	-20.871	0.1594	-15.849	0.2878	-18.449	0.3780	-19.919	0.2381
667.7	-21.404	0.1586	-21.481	0.1266	-21.422	0.1364	-21.331	0.1345	-21.013	0.1608	-15.961	0.2892	-18.575	0.3809	-20.055	0.2399
669.6	-21.552	0.1603	-21.627	0.1275	-21.570	0.1377	-21.475	0.1358	-21.154	0.1621	-16.074	0.2906	-18.702	0.3838	-20.192	0.2417

671.5	-21.700	0.1620	-21.775	0.1284	-21.718	0.1391	-21.619	0.1370	-21.296	0.1635	-16.187	0.2921	-18.829	0.3867	-20.328	0.2435
673.4	-21.849	0.1637	-21.922	0.1294	-21.866	0.1405	-21.764	0.1383	-21.439	0.1648	-16.301	0.2935	-18.956	0.3897	-20.465	0.2453
675.3	-21.997	0.1654	-22.070	0.1303	-22.015	0.1419	-21.910	0.1396	-21.582	0.1662	-16.415	0.2950	-19.084	0.3926	-20.603	0.2471
677.2	-22.147	0.1671	-22.219	0.1313	-22.164	0.1433	-22.055	0.1410	-21.725	0.1676	-16.529	0.2966	-19.212	0.3956	-20.741	0.2490
679.1	-22.297	0.1689	-22.368	0.1323	-22.314	0.1447	-22.202	0.1423	-21.869	0.1689	-16.643	0.2981	-19.341	0.3986	-20.879	0.2508
681.0	-22.447	0.1707	-22.517	0.1333	-22.464	0.1461	-22.348	0.1436	-22.013	0.1703	-16.758	0.2997	-19.470	0.4017	-21.018	0.2527
682.9	-22.598	0.1725	-22.667	0.1343	-22.615	0.1476	-22.495	0.1450	-22.158	0.1718	-16.873	0.3013	-19.599	0.4047	-21.157	0.2546
684.8	-22.749	0.1743	-22.817	0.1353	-22.766	0.1491	-22.643	0.1464	-22.303	0.1732	-16.988	0.3029	-19.729	0.4078	-21.296	0.2565
686.7	-22.900	0.1761	-22.968	0.1363	-22.918	0.1505	-22.791	0.1478	-22.448	0.1746	-17.104	0.3045	-19.859	0.4109	-21.436	0.2584
688.6	-23.052	0.1780	-23.119	0.1374	-23.070	0.1520	-22.939	0.1492	-22.594	0.1761	-17.220	0.3062	-19.989	0.4141	-21.576	0.2604
690.5	-23.205	0.1799	-23.270	0.1384	-23.222	0.1536	-23.088	0.1506	-22.740	0.1775	-17.337	0.3079	-20.120	0.4172	-21.717	0.2623
692.4	-23.358	0.1818	-23.422	0.1395	-23.375	0.1551	-23.237	0.1521	-22.886	0.1790	-17.453	0.3096	-20.251	0.4204	-21.858	0.2643
694.3	-23.511	0.1837	-23.575	0.1405	-23.528	0.1566	-23.387	0.1535	-23.033	0.1805	-17.570	0.3114	-20.382	0.4236	-22.000	0.2663
696.2	-23.665	0.1857	-23.728	0.1416	-23.682	0.1582	-23.537	0.1550	-23.181	0.1819	-17.688	0.3131	-20.514	0.4268	-22.142	0.2683
698.1	-23.820	0.1876	-23.881	0.1427	-23.836	0.1598	-23.687	0.1565	-23.329	0.1834	-17.806	0.3149	-20.647	0.4301	-22.284	0.2703
700.0	-23.974	0.1896	-24.034	0.1438	-23.991	0.1614	-23.838	0.1580	-23.477	0.1850	-17.924	0.3167	-20.779	0.4333	-22.427	0.2723
701.9	-24.130	0.1916	-24.188	0.1450	-24.146	0.1630	-23.989	0.1595	-23.625	0.1865	-18.042	0.3186	-20.912	0.4366	-22.570	0.2744
703.8	-24.285	0.1937	-24.343	0.1461	-24.302	0.1646	-24.141	0.1610	-23.774	0.1880	-18.161	0.3204	-21.046	0.4400	-22.713	0.2764
705.7	-24.442	0.1957	-24.498	0.1472	-24.458	0.1663	-24.293	0.1626	-23.924	0.1896	-18.280	0.3223	-21.180	0.4433	-22.857	0.2785
707.6	-24.598	0.1978	-24.653	0.1484	-24.614	0.1680	-24.446	0.1641	-24.074	0.1911	-18.399	0.3242	-21.314	0.4467	-23.001	0.2806
709.5	-24.755	0.1999	-24.809	0.1496	-24.771	0.1696	-24.599	0.1657	-24.224	0.1927	-18.519	0.3262	-21.448	0.4501	-23.146	0.2827
711.4	-24.913	0.2020	-24.965	0.1507	-24.929	0.1713	-24.753	0.1673	-24.374	0.1943	-18.639	0.3281	-21.583	0.4535	-23.291	0.2849
713.3	-25.071	0.2042	-25.122	0.1519	-25.087	0.1731	-24.907	0.1689	-24.525	0.1958	-18.759	0.3301	-21.719	0.4570	-23.437	0.2870
715.2	-25.229	0.2063	-25.279	0.1531	-25.245	0.1748	-25.061	0.1706	-24.677	0.1975	-18.880	0.3321	-21.854	0.4605	-23.583	0.2892
717.1	-25.388	0.2085	-25.437	0.1544	-25.404	0.1766	-25.216	0.1722	-24.829	0.1991	-19.001	0.3342	-21.990	0.4640	-23.729	0.2914
719.0	-25.548	0.2108	-25.595	0.1556	-25.563	0.1783	-25.371	0.1739	-24.981	0.2007	-19.123	0.3362	-22.127	0.4675	-23.876	0.2936
720.9	-25.707	0.2130	-25.753	0.1568	-25.722	0.1801	-25.527	0.1756	-25.134	0.2023	-19.244	0.3383	-22.264	0.4711	-24.023	0.2958
722.8	-25.868	0.2153	-25.912	0.1581	-25.883	0.1819	-25.683	0.1773	-25.287	0.2040	-19.366	0.3404	-22.401	0.4747	-24.170	0.2980
724.7	-26.029	0.2176	-26.071	0.1593	-26.043	0.1838	-25.840	0.1790	-25.440	0.2056	-19.489	0.3425	-22.539	0.4783	-24.318	0.3003
726.6	-26.190	0.2199	-26.231	0.1606	-26.204	0.1856	-25.997	0.1807	-25.594	0.2073	-19.612	0.3447	-22.677	0.4819	-24.467	0.3025
728.5	-26.352	0.2222	-26.391	0.1619	-26.366	0.1875	-26.154	0.1825	-25.749	0.2090	-19.735	0.3469	-22.815	0.4856	-24.616	0.3048
730.4	-26.514	0.2246	-26.552	0.1632	-26.528	0.1894	-26.312	0.1842	-25.903	0.2107	-19.858	0.3491	-22.954	0.4893	-24.765	0.3071
732.3	-26.676	0.2270	-26.713	0.1646	-26.690	0.1913	-26.471	0.1860	-26.059	0.2124	-19.982	0.3513	-23.093	0.4930	-24.914	0.3095
734.2	-26.840	0.2294	-26.874	0.1659	-26.853	0.1932	-26.629	0.1878	-26.214	0.2142	-20.106	0.3535	-23.233	0.4967	-25.064	0.3118
736.1	-27.003	0.2319	-27.036	0.1672	-27.016	0.1952	-26.789	0.1896	-26.370	0.2159	-20.231	0.3558	-23.373	0.5005	-25.215	0.3142
738.0	-27.167	0.2343	-27.199	0.1686	-27.180	0.1971	-26.948	0.1915	-26.527	0.2176	-20.356	0.3581	-23.513	0.5043	-25.366	0.3165
739.9	-27.332	0.2368	-27.361	0.1700	-27.344	0.1991	-27.108	0.1933	-26.684	0.2194	-20.481	0.3604	-23.654	0.5082	-25.517	0.3189
741.9	-27.497	0.2394	-27.525	0.1714	-27.509	0.2011	-27.269	0.1952	-26.841	0.2212	-20.606	0.3628	-23.795	0.5120	-25.669	0.3214
743.8	-27.662	0.2419	-27.688	0.1728	-27.674	0.2032	-27.430	0.1971	-26.998	0.2230	-20.732	0.3651	-23.936	0.5159	-25.821	0.3238
745.7	-27.828	0.2445	-27.852	0.1742	-27.840	0.2052	-27.592	0.1990	-27.157	0.2248	-20.858	0.3675	-24.078	0.5198	-25.973	0.3262
747.6	-27.995	0.2471	-28.017	0.1756	-28.006	0.2073	-27.754	0.2010	-27.315	0.2266	-20.985	0.3700	-24.221	0.5238	-26.126	0.3287
749.5	-28.162	0.2498	-28.182	0.1771	-28.172	0.2094	-27.916	0.2029	-27.474	0.2284	-21.112	0.3724	-24.363	0.5278	-26.279	0.3312
751.4	-28.329	0.2524	-28.347	0.1785	-28.339	0.2115	-28.079	0.2049	-27.633	0.2303	-21.239	0.3749	-24.506	0.5318	-26.433	0.3337
753.3	-28.497	0.2551	-28.513	0.1800	-28.507	0.2136	-28.242	0.2069	-27.793	0.2321	-21.367	0.3774	-24.650	0.5358	-26.587	0.3363
755.2	-28.665	0.2578	-28.680	0.1815	-28.675	0.2158	-28.406	0.2089	-27.953	0.2340	-21.495	0.3799	-24.794	0.5399	-26.742	0.3388
757.1	-28.834	0.2606	-28.847	0.1830	-28.843	0.2180	-28.570	0.2110	-28.114	0.2359	-21.623	0.3825	-24.938	0.5440	-26.897	0.3414

759.0	-29.003	0.2634	-29.014	0.1845	-29.012	0.2202	-28.735	0.2130	-28.275	0.2378	-21.752	0.3850	-25.083	0.5481	-27.052	0.3440
760.9	-29.173	0.2662	-29.182	0.1860	-29.181	0.2224	-28.900	0.2151	-28.436	0.2397	-21.881	0.3876	-25.228	0.5522	-27.208	0.3466
762.8	-29.343	0.2690	-29.350	0.1876	-29.351	0.2246	-29.065	0.2172	-28.598	0.2416	-22.010	0.3903	-25.373	0.5564	-27.364	0.3492
764.7	-29.514	0.2719	-29.518	0.1891	-29.521	0.2269	-29.231	0.2193	-28.761	0.2435	-22.140	0.3929	-25.519	0.5606	-27.521	0.3519
766.6	-29.685	0.2748	-29.687	0.1907	-29.692	0.2292	-29.398	0.2215	-28.923	0.2455	-22.270	0.3956	-25.665	0.5649	-27.678	0.3546
768.5	-29.857	0.2777	-29.857	0.1923	-29.863	0.2315	-29.565	0.2236	-29.086	0.2475	-22.401	0.3983	-25.812	0.5692	-27.836	0.3573
770.4	-30.029	0.2807	-30.027	0.1939	-30.035	0.2338	-29.732	0.2258	-29.250	0.2494	-22.531	0.4010	-25.959	0.5735	-27.994	0.3600
772.3	-30.202	0.2837	-30.197	0.1955	-30.207	0.2362	-29.900	0.2280	-29.414	0.2514	-22.663	0.4038	-26.106	0.5778	-28.152	0.3627
774.2	-30.375	0.2867	-30.368	0.1972	-30.380	0.2386	-30.068	0.2302	-29.578	0.2534	-22.794	0.4066	-26.254	0.5822	-28.311	0.3655
776.1	-30.549	0.2898	-30.539	0.1988	-30.553	0.2410	-30.237	0.2325	-29.743	0.2555	-22.926	0.4094	-26.402	0.5866	-28.470	0.3682
778.0	-30.723	0.2929	-30.711	0.2005	-30.726	0.2434	-30.406	0.2347	-29.908	0.2575	-23.058	0.4122	-26.551	0.5910	-28.630	0.3710
779.9	-30.897	0.2960	-30.883	0.2022	-30.900	0.2459	-30.576	0.2370	-30.074	0.2595	-23.191	0.4151	-26.700	0.5955	-28.790	0.3739
781.8	-31.072	0.2991	-31.056	0.2039	-31.075	0.2483	-30.746	0.2393	-30.240	0.2616	-23.324	0.4180	-26.850	0.6000	-28.950	0.3767
783.7	-31.248	0.3023	-31.229	0.2056	-31.250	0.2508	-30.916	0.2417	-30.407	0.2637	-23.457	0.4209	-26.999	0.6045	-29.111	0.3796
785.6	-31.424	0.3055	-31.403	0.2073	-31.425	0.2534	-31.087	0.2440	-30.574	0.2658	-23.591	0.4238	-27.150	0.6091	-29.272	0.3825
787.5	-31.601	0.3088	-31.577	0.2091	-31.601	0.2559	-31.259	0.2464	-30.741	0.2679	-23.725	0.4268	-27.300	0.6137	-29.434	0.3854
789.4	-31.778	0.3121	-31.751	0.2109	-31.778	0.2585	-31.431	0.2488	-30.909	0.2700	-23.859	0.4298	-27.451	0.6183	-29.596	0.3883
791.3	-31.955	0.3154	-31.926	0.2126	-31.955	0.2611	-31.603	0.2513	-31.077	0.2722	-23.994	0.4329	-27.603	0.6230	-29.759	0.3913
793.2	-32.133	0.3187	-32.101	0.2144	-32.132	0.2637	-31.776	0.2537	-31.246	0.2743	-24.129	0.4359	-27.755	0.6277	-29.922	0.3942
795.1	-32.312	0.3221	-32.277	0.2163	-32.310	0.2664	-31.949	0.2562	-31.415	0.2765	-24.265	0.4390	-27.907	0.6324	-30.086	0.3972
797.0	-32.491	0.3255	-32.453	0.2181	-32.488	0.2691	-32.123	0.2587	-31.584	0.2787	-24.401	0.4421	-28.060	0.6372	-30.249	0.4003
798.9	-32.670	0.3290	-32.630	0.2199	-32.667	0.2718	-32.297	0.2612	-31.754	0.2809	-24.537	0.4453	-28.213	0.6420	-30.414	0.4033
800.8	-32.850	0.3325	-32.807	0.2218	-32.846	0.2745	-32.472	0.2638	-31.925	0.2831	-24.673	0.4484	-28.366	0.6468	-30.578	0.4064
802.7	-33.031	0.3360	-32.985	0.2237	-33.026	0.2773	-32.647	0.2663	-32.095	0.2853	-24.810	0.4517	-28.520	0.6517	-30.744	0.4095
804.6	-33.212	0.3396	-33.163	0.2256	-33.206	0.2801	-32.823	0.2689	-32.267	0.2876	-24.948	0.4549	-28.674	0.6566	-30.909	0.4126
806.5	-33.393	0.3432	-33.342	0.2275	-33.387	0.2829	-32.999	0.2715	-32.438	0.2898	-25.085	0.4582	-28.829	0.6616	-31.075	0.4157
808.4	-33.575	0.3468	-33.521	0.2295	-33.568	0.2857	-33.175	0.2742	-32.610	0.2921	-25.223	0.4614	-28.984	0.6665	-31.242	0.4189
810.3	-33.757	0.3505	-33.700	0.2314	-33.750	0.2886	-33.352	0.2769	-32.783	0.2944	-25.362	0.4648	-29.140	0.6716	-31.409	0.4221
812.2	-33.940	0.3542	-33.880	0.2334	-33.932	0.2915	-33.530	0.2796	-32.956	0.2967	-25.501	0.4681	-29.295	0.6766	-31.576	0.4253
814.1	-34.124	0.3580	-34.061	0.2354	-34.115	0.2944	-33.708	0.2823	-33.129	0.2991	-25.640	0.4715	-29.452	0.6817	-31.744	0.4285
816.0	-34.308	0.3618	-34.241	0.2374	-34.298	0.2974	-33.886	0.2850	-33.303	0.3014	-25.779	0.4749	-29.609	0.6868	-31.912	0.4318
817.9	-34.492	0.3656	-34.423	0.2394	-34.482	0.3003	-34.065	0.2878	-33.477	0.3038	-25.919	0.4784	-29.766	0.6920	-32.081	0.4351
819.8	-34.677	0.3695	-34.605	0.2415	-34.666	0.3034	-34.245	0.2906	-33.652	0.3061	-26.060	0.4819	-29.923	0.6972	-32.250	0.4384
821.7	-34.863	0.3734	-34.787	0.2436	-34.850	0.3064	-34.424	0.2935	-33.827	0.3085	-26.200	0.4854	-30.081	0.7024	-32.419	0.4417
823.6	-35.049	0.3773	-34.970	0.2456	-35.035	0.3095	-34.605	0.2963	-34.003	0.3109	-26.341	0.4889	-30.240	0.7077	-32.589	0.4451
825.5	-35.235	0.3813	-35.153	0.2478	-35.221	0.3126	-34.786	0.2992	-34.179	0.3134	-26.483	0.4925	-30.398	0.7130	-32.759	0.4485
827.4	-35.422	0.3853	-35.336	0.2499	-35.407	0.3157	-34.967	0.3021	-34.355	0.3158	-26.624	0.4961	-30.558	0.7183	-32.930	0.4519
829.3	-35.609	0.3894	-35.520	0.2520	-35.594	0.3189	-35.149	0.3050	-34.532	0.3183	-26.767	0.4997	-30.717	0.7237	-33.101	0.4553
831.2	-35.797	0.3935	-35.705	0.2542	-35.781	0.3220	-35.331	0.3080	-34.709	0.3208	-26.909	0.5034	-30.877	0.7291	-33.273	0.4588
833.1	-35.986	0.3977	-35.890	0.2564	-35.968	0.3253	-35.513	0.3110	-34.887	0.3233	-27.052	0.5071	-31.038	0.7346	-33.445	0.4623
835.0	-36.175	0.4019	-36.076	0.2586	-36.156	0.3285	-35.697	0.3140	-35.065	0.3258	-27.195	0.5109	-31.198	0.7401	-33.618	0.4658
836.9	-36.364	0.4061	-36.262	0.2608	-36.345	0.3318	-35.880	0.3171	-35.244	0.3283	-27.339	0.5146	-31.360	0.7456	-33.791	0.4693
838.8	-36.554	0.4104	-36.448	0.2630	-36.534	0.3351	-36.064	0.3202	-35.423	0.3308	-27.483	0.5184	-31.521	0.7512	-33.964	0.4729
840.7	-36.745	0.4147	-36.635	0.2653	-36.723	0.3385	-36.249	0.3233	-35.602	0.3334	-27.627	0.5223	-31.683	0.7568	-34.138	0.4765
842.6	-36.936	0.4191	-36.822	0.2676	-36.913	0.3418	-36.434	0.3264	-35.782	0.3360	-27.772	0.5261	-31.846	0.7624	-34.312	0.4801
844.5	-37.127	0.4235	-37.010	0.2699	-37.104	0.3453	-36.619	0.3296	-35.962	0.3386	-27.917	0.5300	-32.009	0.7681	-34.487	0.4838

846.4	-37.319	0.4279	-37.198	0.2722	-37.295	0.3487	-36.805	0.3328	-36.143	0.3412	-28.063	0.5340	-32.172	0.7739	-34.662	0.4875
848.3	-37.512	0.4324	-37.387	0.2746	-37.486	0.3522	-36.992	0.3360	-36.324	0.3439	-28.209	0.5380	-32.336	0.7796	-34.838	0.4912
850.2	-37.705	0.4369	-37.576	0.2769	-37.678	0.3557	-37.179	0.3393	-36.506	0.3465	-28.355	0.5420	-32.500	0.7855	-35.014	0.4949
852.1	-37.899	0.4415	-37.766	0.2793	-37.871	0.3592	-37.366	0.3426	-36.688	0.3492	-28.502	0.5460	-32.665	0.7913	-35.191	0.4987
854.0	-38.093	0.4462	-37.956	0.2817	-38.063	0.3628	-37.554	0.3459	-36.871	0.3519	-28.649	0.5501	-32.830	0.7972	-35.367	0.5025
855.9	-38.287	0.4508	-38.147	0.2842	-38.257	0.3664	-37.743	0.3492	-37.054	0.3546	-28.796	0.5542	-32.995	0.8032	-35.545	0.5063
857.8	-38.482	0.4556	-38.338	0.2866	-38.451	0.3701	-37.931	0.3526	-37.237	0.3573	-28.944	0.5584	-33.161	0.8091	-35.723	0.5101
859.7	-38.678	0.4603	-38.530	0.2891	-38.645	0.3737	-38.121	0.3560	-37.421	0.3601	-29.092	0.5626	-33.327	0.8152	-35.901	0.5140
861.6	-38.874	0.4651	-38.722	0.2916	-38.840	0.3775	-38.311	0.3595	-37.605	0.3628	-29.241	0.5668	-33.494	0.8212	-36.080	0.5179
863.5	-39.071	0.4700	-38.914	0.2941	-39.036	0.3812	-38.501	0.3630	-37.790	0.3656	-29.390	0.5711	-33.661	0.8273	-36.259	0.5219
865.4	-39.268	0.4749	-39.107	0.2966	-39.232	0.3850	-38.692	0.3665	-37.975	0.3684	-29.539	0.5754	-33.829	0.8335	-36.438	0.5258
867.3	-39.466	0.4799	-39.301	0.2992	-39.428	0.3888	-38.883	0.3700	-38.161	0.3712	-29.689	0.5797	-33.997	0.8397	-36.619	0.5298
869.2	-39.664	0.4849	-39.495	0.3018	-39.625	0.3927	-39.075	0.3736	-38.347	0.3741	-29.839	0.5841	-34.165	0.8459	-36.799	0.5339
871.1	-39.862	0.4899	-39.689	0.3044	-39.823	0.3966	-39.267	0.3772	-38.533	0.3770	-29.989	0.5885	-34.334	0.8522	-36.980	0.5379
873.0	-40.062	0.4951	-39.884	0.3070	-40.020	0.4005	-39.460	0.3808	-38.720	0.3798	-30.140	0.5930	-34.503	0.8585	-37.161	0.5420
874.9	-40.261	0.5002	-40.080	0.3097	-40.219	0.4044	-39.653	0.3845	-38.908	0.3827	-30.292	0.5975	-34.673	0.8649	-37.343	0.5461
876.8	-40.462	0.5054	-40.275	0.3124	-40.418	0.4085	-39.847	0.3882	-39.095	0.3857	-30.443	0.6020	-34.843	0.8713	-37.526	0.5503
878.7	-40.663	0.5107	-40.472	0.3151	-40.617	0.4125	-40.041	0.3920	-39.284	0.3886	-30.595	0.6066	-35.013	0.8778	-37.708	0.5544
880.6	-40.864	0.5160	-40.669	0.3178	-40.817	0.4166	-40.236	0.3958	-39.472	0.3916	-30.748	0.6112	-35.184	0.8843	-37.891	0.5587
882.5	-41.066	0.5214	-40.866	0.3206	-41.018	0.4207	-40.431	0.3996	-39.662	0.3945	-30.901	0.6158	-35.356	0.8908	-38.075	0.5629
884.4	-41.268	0.5268	-41.064	0.3233	-41.219	0.4248	-40.627	0.4034	-39.851	0.3976	-31.054	0.6205	-35.528	0.8974	-38.259	0.5672
886.3	-41.471	0.5322	-41.262	0.3261	-41.420	0.4290	-40.823	0.4073	-40.041	0.4006	-31.207	0.6253	-35.700	0.9041	-38.444	0.5715
888.2	-41.674	0.5378	-41.461	0.3290	-41.622	0.4333	-41.019	0.4112	-40.232	0.4036	-31.361	0.6300	-35.872	0.9108	-38.629	0.5758
890.1	-41.878	0.5433	-41.660	0.3318	-41.825	0.4375	-41.217	0.4152	-40.423	0.4067	-31.516	0.6348	-36.045	0.9175	-38.814	0.5802
892.0	-42.083	0.5490	-41.859	0.3347	-42.028	0.4419	-41.414	0.4192	-40.614	0.4098	-31.671	0.6397	-36.219	0.9243	-39.000	0.5846
893.9	-42.288	0.5546	-42.060	0.3376	-42.231	0.4462	-41.612	0.4232	-40.806	0.4129	-31.826	0.6446	-36.393	0.9311	-39.186	0.5890
895.8	-42.493	0.5604	-42.260	0.3405	-42.435	0.4506	-41.811	0.4273	-40.998	0.4160	-31.981	0.6495	-36.567	0.9380	-39.373	0.5935
897.7	-42.699	0.5662	-42.461	0.3435	-42.640	0.4550	-42.010	0.4314	-41.191	0.4192	-32.137	0.6545	-36.742	0.9449	-39.560	0.5980
899.6	-42.906	0.5720	-42.663	0.3464	-42.845	0.4595	-42.210	0.4355	-41.384	0.4223	-32.294	0.6595	-36.917	0.9519	-39.748	0.6025
901.5	-43.113	0.5779	-42.865	0.3494	-43.050	0.4640	-42.410	0.4397	-41.578	0.4255	-32.450	0.6646	-37.093	0.9589	-39.936	0.6071
903.4	-43.321	0.5839	-43.068	0.3525	-43.256	0.4686	-42.610	0.4439	-41.772	0.4287	-32.607	0.6697	-37.269	0.9660	-40.125	0.6117
905.3	-43.529	0.5899	-43.271	0.3555	-43.463	0.4732	-42.811	0.4482	-41.966	0.4320	-32.765	0.6749	-37.446	0.9731	-40.314	0.6163
907.2	-43.738	0.5960	-43.474	0.3586	-43.670	0.4778	-43.013	0.4524	-42.161	0.4352	-32.923	0.6801	-37.622	0.9803	-40.504	0.6210
909.1	-43.947	0.6021	-43.678	0.3617	-43.878	0.4825	-43.215	0.4568	-42.357	0.4385	-33.081	0.6853	-37.800	0.9875	-40.694	0.6257
911.0	-44.157	0.6083	-43.883	0.3648	-44.086	0.4872	-43.418	0.4611	-42.553	0.4418	-33.240	0.6906	-37.978	0.9948	-40.884	0.6305
912.9	-44.367	0.6146	-44.088	0.3680	-44.295	0.4920	-43.621	0.4656	-42.749	0.4451	-33.399	0.6959	-38.156	1.0021	-41.075	0.6352
914.8	-44.578	0.6209	-44.293	0.3712	-44.504	0.4968	-43.824	0.4700	-42.946	0.4485	-33.559	0.7013	-38.335	1.0095	-41.266	0.6400
916.7	-44.789	0.6273	-44.499	0.3744	-44.713	0.5017	-44.028	0.4745	-43.143	0.4518	-33.719	0.7067	-38.514	1.0169	-41.458	0.6449
918.6	-45.001	0.6337	-44.706	0.3776	-44.924	0.5066	-44.233	0.4790	-43.341	0.4552	-33.879	0.7122	-38.693	1.0244	-41.650	0.6498
920.5	-45.214	0.6402	-44.913	0.3809	-45.134	0.5115	-44.438	0.4836	-43.539	0.4586	-34.040	0.7177	-38.873	1.0319	-41.843	0.6547
922.4	-45.427	0.6468	-45.120	0.3842	-45.346	0.5165	-44.644	0.4882	-43.737	0.4621	-34.201	0.7233	-39.054	1.0395	-42.036	0.6597
924.3	-45.640	0.6534	-45.328	0.3875	-45.557	0.5216	-44.850	0.4929	-43.936	0.4655	-34.362	0.7289	-39.235	1.0471	-42.230	0.6646
926.2	-45.854	0.6601	-45.537	0.3909	-45.770	0.5266	-45.056	0.4976	-44.136	0.4690	-34.524	0.7346	-39.416	1.0548	-42.424	0.6697
928.1	-46.069	0.6668	-45.745	0.3943	-45.982	0.5318	-45.263	0.5023	-44.336	0.4725	-34.687	0.7403	-39.598	1.0625	-42.619	0.6747
930.0	-46.284	0.6737	-45.955	0.3977	-46.196	0.5370	-45.471	0.5071	-44.536	0.4761	-34.849	0.7460	-39.780	1.0703	-42.814	0.6798
931.9	-46.500	0.6805	-46.165	0.4011	-46.409	0.5422	-45.679	0.5119	-44.737	0.4796	-35.012	0.7518	-39.962	1.0781	-43.009	0.6850

933.8	-46.716	0.6875	-46.375	0.4046	-46.624	0.5474	-45.888	0.5168	-44.939	0.4832	-35.176	0.7577	-40.146	1.0860	-43.205	0.6902
935.7	-46.933	0.6945	-46.586	0.4081	-46.839	0.5528	-46.097	0.5217	-45.140	0.4868	-35.340	0.7636	-40.329	1.0940	-43.401	0.6954
937.6	-47.150	0.7016	-46.797	0.4116	-47.054	0.5581	-46.306	0.5267	-45.343	0.4904	-35.504	0.7695	-40.513	1.1020	-43.598	0.7006
939.5	-47.368	0.7087	-47.009	0.4152	-47.270	0.5636	-46.516	0.5317	-45.545	0.4941	-35.669	0.7756	-40.697	1.1101	-43.796	0.7059
941.5	-47.586	0.7159	-47.221	0.4188	-47.486	0.5690	-46.727	0.5367	-45.748	0.4977	-35.834	0.7816	-40.882	1.1182	-43.993	0.7113
943.4	-47.805	0.7232	-47.434	0.4224	-47.703	0.5745	-46.938	0.5418	-45.952	0.5014	-36.000	0.7877	-41.067	1.1264	-44.192	0.7167
945.3	-48.025	0.7305	-47.648	0.4260	-47.921	0.5801	-47.150	0.5469	-46.156	0.5052	-36.166	0.7939	-41.253	1.1346	-44.391	0.7221
947.2	-48.245	0.7380	-47.861	0.4297	-48.139	0.5857	-47.362	0.5521	-46.360	0.5089	-36.332	0.8001	-41.439	1.1429	-44.590	0.7275
949.1	-48.466	0.7454	-48.076	0.4334	-48.358	0.5914	-47.575	0.5573	-46.565	0.5127	-36.499	0.8063	-41.626	1.1512	-44.789	0.7330
951.0	-48.687	0.7530	-48.291	0.4372	-48.577	0.5971	-47.788	0.5626	-46.771	0.5165	-36.666	0.8126	-41.813	1.1596	-44.990	0.7386
952.9	-48.909	0.7606	-48.506	0.4409	-48.796	0.6029	-48.001	0.5679	-46.977	0.5203	-36.834	0.8190	-42.000	1.1681	-45.190	0.7441
954.8	-49.131	0.7683	-48.722	0.4447	-49.016	0.6087	-48.216	0.5733	-47.183	0.5242	-37.002	0.8254	-42.188	1.1766	-45.391	0.7497
956.7	-49.354	0.7761	-48.938	0.4486	-49.237	0.6146	-48.430	0.5787	-47.390	0.5280	-37.170	0.8319	-42.377	1.1852	-45.593	0.7554
958.6	-49.577	0.7839	-49.155	0.4525	-49.458	0.6205	-48.645	0.5842	-47.597	0.5319	-37.339	0.8384	-42.566	1.1938	-45.795	0.7611
960.5	-49.801	0.7918	-49.372	0.4564	-49.680	0.6265	-48.861	0.5897	-47.805	0.5359	-37.508	0.8450	-42.755	1.2025	-45.997	0.7668
962.4	-50.026	0.7998	-49.590	0.4603	-49.902	0.6325	-49.077	0.5952	-48.013	0.5398	-37.678	0.8517	-42.945	1.2113	-46.200	0.7726
964.3	-50.251	0.8079	-49.808	0.4643	-50.125	0.6386	-49.294	0.6008	-48.222	0.5438	-37.848	0.8583	-43.135	1.2201	-46.404	0.7785
966.2	-50.476	0.8160	-50.027	0.4683	-50.348	0.6447	-49.511	0.6065	-48.431	0.5478	-38.018	0.8651	-43.325	1.2289	-46.608	0.7843
968.1	-50.702	0.8242	-50.246	0.4723	-50.572	0.6509	-49.729	0.6122	-48.640	0.5519	-38.189	0.8719	-43.516	1.2379	-46.812	0.7902
970.0	-50.929	0.8325	-50.466	0.4764	-50.797	0.6572	-49.947	0.6180	-48.850	0.5559	-38.361	0.8788	-43.708	1.2469	-47.017	0.7962
971.9	-51.156	0.8409	-50.686	0.4805	-51.022	0.6635	-50.166	0.6238	-49.061	0.5600	-38.532	0.8857	-43.900	1.2559	-47.222	0.8022
973.8	-51.384	0.8493	-50.907	0.4846	-51.247	0.6699	-50.386	0.6296	-49.272	0.5641	-38.705	0.8927	-44.092	1.2651	-47.428	0.8082
975.7	-51.613	0.8579	-51.128	0.4888	-51.473	0.6763	-50.605	0.6356	-49.483	0.5683	-38.877	0.8997	-44.285	1.2742	-47.634	0.8143
977.6	-51.841	0.8665	-51.350	0.4930	-51.700	0.6828	-50.826	0.6415	-49.695	0.5724	-39.050	0.9068	-44.479	1.2835	-47.841	0.8204
979.5	-52.071	0.8751	-51.572	0.4973	-51.927	0.6893	-51.047	0.6475	-49.907	0.5766	-39.223	0.9139	-44.672	1.2928	-48.048	0.8266
981.4	-52.301	0.8839	-51.795	0.5015	-52.154	0.6959	-51.268	0.6536	-50.120	0.5808	-39.397	0.9211	-44.867	1.3022	-48.256	0.8328
983.3	-52.532	0.8927	-52.019	0.5058	-52.383	0.7025	-51.490	0.6597	-50.334	0.5851	-39.572	0.9284	-45.061	1.3116	-48.464	0.8391
985.2	-52.763	0.9017	-52.242	0.5102	-52.611	0.7092	-51.712	0.6659	-50.547	0.5894	-39.746	0.9357	-45.256	1.3211	-48.673	0.8454
987.1	-52.995	0.9107	-52.467	0.5146	-52.840	0.7160	-51.935	0.6721	-50.762	0.5937	-39.921	0.9431	-45.452	1.3307	-48.882	0.8518
989.0	-53.227	0.9197	-52.692	0.5190	-53.070	0.7228	-52.159	0.6784	-50.976	0.5980	-40.097	0.9506	-45.648	1.3403	-49.091	0.8582
990.9	-53.460	0.9289	-52.917	0.5235	-53.301	0.7297	-52.383	0.6847	-51.191	0.6024	-40.273	0.9581	-45.845	1.3500	-49.301	0.8646
992.8	-53.693	0.9382	-53.143	0.5280	-53.531	0.7367	-52.607	0.6911	-51.407	0.6068	-40.449	0.9657	-46.042	1.3598	-49.512	0.8711
994.7	-53.927	0.9475	-53.369	0.5325	-53.763	0.7437	-52.832	0.6976	-51.623	0.6112	-40.626	0.9733	-46.239	1.3696	-49.723	0.8777
996.6	-54.162	0.9569	-53.596	0.5371	-53.995	0.7508	-53.058	0.7041	-51.840	0.6157	-40.803	0.9810	-46.437	1.3795	-49.935	0.8843
998.5	-54.397	0.9664	-53.823	0.5417	-54.227	0.7579	-53.284	0.7106	-52.057	0.6201	-40.981	0.9888	-46.635	1.3894	-50.147	0.8909

Supporting Information References

1. Fokin, D.A. *et al.* Electronic growth of Pb on the vicinal Si surface. *Phys. Status Solidi C* **7**, 165-168 (2010).
2. Su, W.B. *et al.* Correlation between quantized electronic states and oscillatory thickness relaxations of 2D Pb islands on Si (111)-(7× 7) surfaces. *Phys. Rev. Lett.* **86**, 5116 (2001).
3. Kern, R., Le Lay, G. and J.J. Metois. *Current Topics in Materials Science* **3**, 131, ed. by E. Kaldis (1979).
4. Bromann, K. et al. Interlayer mass transport in homoepitaxial and heteroepitaxial metal growth. *Phys. Rev. Lett.* **75**, 677 (1995).
5. Sette, F. et al. Coverage and chemical dependence of adsorbate-induced bond weakening in metal substrate surfaces. *Phys. Rev. Lett.* **61**, 1384 (1988).
6. Baski, A.A. and Fuchs, H. Epitaxial growth of silver on mica as studied by AFM and STM. *Surf. Sci.* **313**, 275-288 (1994).
7. Davey, W. P. Precision measurements of the lattice constants of twelve common metals. P*hys. Rev.* **25**, 753 (1925).
8. Zaumseil, P. High-resolution characterization of the forbidden Si 200 and Si 222 reflections. *J. Appl. Crystallogr.* **48**, 528 (2015).
9. Durand, O. et al. Interpretation of the two-components observed in high resolution X-ray diffraction ω scan peaks for mosaic ZnO thin films grown on c-sapphire substrates using pulsed laser deposition. *Thin Solid Films* **519**, 6369 (2011).
10. High, A.A. *et al.* Visible-frequency hyperbolic metasurface. *Nature* **522**, 192 (2015).
11. Park, J.H. *et al.* Single-Crystalline Silver Films for Plasmonics. *Adv. Mater.* **24**, 3988-3992 (2012).
12. Brendel R, Bormann D. An infrared dielectric function model for amorphous solids. *Journal of Appl. Phys.* **71**, 1–6 (1992).
13. Johnson, P.B. and Christy, R.W. Optical constants of the noble metals. *Phys. Rev. B* **6**, 4370 (1972).
14. Pinchuk, A., Kreibig, U., Hilger A. Optical properties of metallic nanoparticles: influence of interface effects and interband transitions. *Surf. Sci.* **557**, 269–280 (2004).
15. Rodionov, I.A. et al. Crystalline structure dependence on optical properties of silver thin film over time. *2017 Progress In Electromagnetics Research Symposium — Spring (PIERS)*, 1497-1502 (2017).
16. Kreibig, U. et al. Interfaces in nanostructures: optical investigations on cluster-matter. *Nanostructured Materials*, 11, 1335-1342 (1999).

17. Baburin, A. S. et al. Toward a theoretically limited SPP propagation length above two hundred microns on an ultra-smooth silver surface. *Opt. Mater. Express* **8**,11: 3254-3261 (2018).
18. Wu, Y. *et al.* Intrinsic optical properties and enhanced plasmonic response of epitaxial silver. *Adv. Mater.* **26**, 6106-6110 (2014).
